# Muslim religious leaders’ views of climate change: An international survey

**DOI:** 10.1371/journal.pone.0353201

**Published:** 2026-08-25

**Authors:** Catherine E. Bowen, Konrad Pędziwiatr, Marcin Stonawski, Vegard Skirbekk

**Affiliations:** 1 Department of Demography, University of Vienna, Austria; 2 Center for Advanced Studies of Population and Religion (CASPAR), Krakow University of Economics, Krakow, Poland; 3 International Institute for Applied Systems Analysis (IIASA), Laxenburg, Austria; 4 Department of Psychology, University of Oslo, Oslo, Norway; 5 Centre for Fertility and Health, Norwegian Institute of Public Health, Oslo, Norway; Federal University Otuoke, NIGERIA

## Abstract

Religious leaders have unique potential to motivate individuals and governments to address climate change. Nevertheless, there is currently a lack of systematic information on religious leaders’ views of climate change and their willingness to raise awareness and encourage climate mitigation behaviors. We present results from an exploratory global survey of Muslim religious leaders (*N* = 150 from 29 countries; e.g., imams, religious teachers, students of religious seminaries). Nearly all respondents agreed that the climate is changing (98.6%), and about half agreed that climate change is mainly or entirely caused by humans (48.7%). Nearly half (48.0%) agreed that climate change is *already* substantially harming people. Most respondents indicated that Muslim religious leaders and the Muslim religious community should do more to address climate change, and that Islam offers a set of values and principles that motivate environmental protection and climate action. Respondents reported that they sometimes addressed climate change and other environmental topics in their religious work. They reported high willingness to support government spending on technologies that curb emissions and the effects of climate change; the implementation of new technologies to promote greener production and consumption; and reductions in consumption. There was lower willingness to promote dietary changes and reduced meat consumption; vegetarianism; or limits on population growth and family size. These preliminary findings suggest that, although many respondents are not regularly addressing climate change in their religious work, Muslim religious leaders may have substantial potential to promote climate change awareness and action.

## Introduction

Religious leaders have a distinct capacity to raise awareness about climate change and encourage environmentally responsible behavior. Currently, more than 80% of the global population is affiliated with a religion [1]. Theoretical and empirical work suggests that individuals’ climate beliefs and behavior are influenced by their morals [2,3], values [4], and the norms and shared beliefs of the groups with which they identify [5]. By linking environmental concern with the values and principles of their faith, religious leaders can provide a moral framework for climate action [6], shaping how followers perceive and respond to climate issues and the degree to which climate concern becomes a shared group norm. Religious leaders often have a “captive audience” (e.g., members of a congregation; students at a school) and access to financial, organizational, and human resources that could potentially be mobilized. They have contact with and influence diverse communities, and some wield significant political clout. Religious leaders may be especially well poised to influence conservative groups, who tend to be relatively skeptical of climate change [7], religious [8], and wary of science and climate science in particular [8]. At the same time, religious belief is not necessarily at odds with confidence in science [9]. There is also broad consensus that elites influence public opinion about climate change and mitigation efforts [10,11]. Experimental [12–14] and longitudinal [15] research confirms that environmental attitudes and behaviors can be shaped by the views of religious elites. Together, this body of work suggests that religious leaders may be important actors in shaping climate attitudes and behaviors among the broader public. Examining religious leaders’ own climate beliefs and behavioral intentions is therefore an important area of inquiry.

Currently, however, little is known about religious leaders’ views on climate change. A number of religious organizations and (groups of) religious leaders have published formal statements about climate change, including Pope Francis [16] and Tenzin Gyatso, the 14th Dalai Lama [17] [for a review, see 18]. Several qualitative studies have explored how religious actors encourage environmental action [e.g., 19,20]. However, neither the statements by individual figures nor the findings from qualitative research provide insight into the views of the broader population of religious leaders. To date, just a handful of surveys have systematically assessed the climate views of religious leaders. A recent study based on a nationally representative sample of U.S. religious leaders (*N* = 1,600), most of whom were Christian, found that nearly 90% believed in anthropogenic climate change to some degree, yet roughly half had never discussed it with their congregation [14]. Earlier surveys of U.S. Christian clergy provide additional evidence on environmental engagement and denominational differences. In a 1998 survey of U.S. Christian clergy, 46% of respondents reported that they addressed the environment in their sermons either often or very often [21], while other surveys of Christian clergy and religious activists in the United States found variation in environmental concern across Christian denominations [22]. More recently, a 2019 telephone survey of *N* = 1,000 U.S. Protestant pastors found that 53% believed that global warming is real and manmade [23]. Beyond the United States, an online survey about the COVID-19 pandemic of *N* = 1223 religious leaders of multiple faiths primarily from sub-Saharan Africa, the Middle East and Northern Africa (MENA), Europe, and the Americas found that nearly half (47.3%) of respondents agreed that strengthening environmental protection in the wake of the pandemic was extremely important [24]. While these studies provide important insights, the evidence is primarily limited to Christian leaders in the United States and a narrow range of climate-related views.

This study provides descriptive, quantitative evidence on Muslim religious leaders’ beliefs about the causes and consequences of climate change, their perceptions of the role of Muslim leaders in addressing it, and their willingness to personally adopt and promote climate mitigation behaviors. Our aim is to provide an initial empirical assessment of Muslim religious leaders’ climate-related views rather than test specific theoretical predictions. Accordingly, we use research suggesting the potential role of religious leaders in shaping climate-related beliefs and behaviors to contextualize the study rather than to derive formal hypotheses. Few studies have examined religious leaders’ views on climate change, and most existing research has been either qualitative or focused primarily on Christian leaders. By analyzing survey data from Muslim religious leaders, this study helps address an important gap in the literature.

Examining the climate views of Muslim leaders is particularly important because Islam is the world’s second-largest and fastest-growing religion. According to demographic projections, the proportion of Muslims in the global population is expected to rise from 24.1% in 2015 (1.75 billion persons) to 31.1% in 2060 (2.99 billion persons) [1]. The majority of Muslims live in Central Africa, MENA, and Southeast Asia. Many countries in these regions are highly vulnerable to climate change. Fifteen of the approximately 50 Muslim-majority countries rank in the top quarter of climate-vulnerable countries globally [25], and the countries in the MENA region are particularly vulnerable to water scarcity that is expected to worsen with climate change [26]. At the same time, many Muslim-majority countries are also major contributors to climate change through fossil fuel production and deforestation. Islam is the dominant religion in several major oil producing countries, such as Saudi Arabia, Iran, Nigeria, Kuwait and Iraq. Indonesia, the world’s most populous Muslim country, has also played a significant role in global tropical deforestation; between 2010 and 2014, Indonesia was responsible for nearly 14% of global tropical deforestation, making it the world’s second biggest tropical deforester behind Brazil [27]. These trends underscore the importance of understanding how Muslim religious leaders perceive climate change and their potential role in addressing it.

Our study is based on a survey of a geographically, denominationally, and demographically diverse convenience sample of Muslim religious leaders, including imams and muftis, religious teachers, chaplains, students at theological seminaries, community leaders, and village elders. A technical report describing the survey is available elsewhere [28]. In this article, we reframe the results for an academic audience, provide a more in-depth and formalized analysis, and present new results.

## Materials and methods

### Sample

The final sample consisted of *N* = 150 respondents from 29 countries. Respondents were 22–77 years old (*M* = 42.77, *SD* = 13.16). Most were men (78.7%) and had a university or advanced degree (76.3%). The respondents consisted primarily of imams (42.7%), religious teachers (28.0%), and community leaders (21.3%). The sample included both Sunni and Shia leaders. Table 1 displays the descriptive statistics of the sample.

**Table 1:** Descriptive characteristics of the sample (N = 150 Muslim religious leaders).

World region and country of residence		n	%
Western Europe and United States of America (n = 32, 21.3%)	Austria	1	0.7
	Belgium	1	0.7
	France	1	0.7
	Germany	1	0.7
	Italy	1	0.7
	Poland	2	1.3
	Sweden	2	1.3
	United Kingdom	20	13.3
	United States of America	3	2.0
post-Soviet (n=17, 11.3%)	Russian Federation	10	6.7
	Ukraine	7	4.7
Middle East and North Africa (n = 44, 29.3%)	Occupied Palestinian Territory	3	2.0
	Iran (Islamic Republic of)	1	0.7
	Iraq	15	10.0
	Jordan	1	0.7
	Saudi Arabia	1	0.7
	Tunisia	22	14.7
	Egypt	1	0.7
sub-Saharan Africa (n=28, 18.7%)	Congo	1	0.7
	Ghana	2	1.3
	Kenya	4	2.7
	Malawi	12	8.0
	Niger	3	2.0
	Somalia	6	4.0
Asia (n = 28, 18.7%)	Bangladesh	4	2.7
	India	14	9.3
	Indonesia	1	0.7
	Pakistan	7	4.7
	Philippines	2	1.3
Missing		1	0.7
**Age**			
Under 30		22	14.7
30–60 years		102	68.0
60+		18	12.0
Missing		8	5.3
**Gender**			
Male		118	78.7
Female		32	21.3
**Education**			
Primary or less		2	1.3
Lower secondary		6	4.0
Upper secondary		13	8.7
Postsecondary, no university certificate		9	6.0
Bachelor’s or equivalent		41	27.3
Master’s or equivalent		48	32.0
PhD or equivalent		30	20.0
Missing		1	0.7
**Type of settlement**			
City above 1 million people		64	42.7
City 300 thousand – 1 million people		38	25.3
Town 1,000–300 thousand people		36	24.0
Village 100–1 thousand people		9	6.0
Hamlet (below 100 people)		1	0.7
Missing		2	1.3
**Marital status**			
Married, living together with spouse		114	76.0
Married, living separated from spouse		3	2.0
Single		28	18.7
Divorced		3	2.0
Widowed		2	1.3
**Madhab (school of jurisprudence)**			
Hanafi		47	31.3
Maliki		36	24.0
Shafi’i		44	29.3
Hanbali		7	4.7
Ja’fari		5	3.3
Zaidi		3	2.0
Other		8	5.3
**Formal religious training**			
Yes		95	63.3
No		54	36.0
Missing		1	0.7
**Leadership role**			
Imam		64	42.7
Teacher		42	28.0
Community leader		32	21.3
Student of establishment of religious education		15	10.0
Village elder		7	4.7
Chaplain		3	2.0
Other		27	18.0
**Number of weekly contacts through religious work**			
Below 10		17	11.3
10-100		48	32.0
100−1,000		54	36.0
1,000-10,000		13	8.7
More than 10,000		8	5.3
Missing		10	6.7
**Mode of data collection**			
Offline		71	47.3
Online		79	52.6

### Procedure

The study was initiated by the Humanitarian Academy for Development and conducted in line with their internal research governance and ethics procedures. Data was collected in July through October, 2018. An offline, paper-based questionnaire was developed in English and Arabic. A preliminary version of the questionnaire was pre-tested in a pilot study with 10 Muslim leaders in Europe. The final questionnaire consisted of 40 items. To increase the potential sample size, we subsequently developed an online version of the survey and translated the survey into additional languages. English served as the master version, from which the questionnaire was forward-translated into Arabic, French, Russian, and Turkish with the assistance of bilingual collaborators. No back-translation or formal validation procedures were employed.

Participants were recruited using a mixture of purposive and snowball methods. In purposive sampling, participants are deliberately selected based on their relevance to the research question, whereas in snowball sampling the sample is generated through referrals from one participant to another. We recruited participants by sending e-mails to various Muslim organizations who agreed to promote the study in their networks (e.g., Muslim Council of Britain, Muslim Religious Union, Egyptian Dar al-Ifta and Al-Azhar, Tunisian Ministry of Religious Affairs); via pollsters collecting data in local mosques and Islamic institutions (in, e.g., Iraq, India, and the Occupied Palestinian Territory), and via the staff of the Humanitarian Academy for Development and Islamic Relief Worldwide in locations across Africa, Asia and Europe.

After reading a detailed description of the study and providing informed consent, respondents completed either the offline (47.3%) or online (52.6%) questionnaire, depending on local logistical constraints, recruitment pathways, and respondent accessibility. Because recruitment strategies varied across contexts, survey mode was not randomly assigned and was unevenly distributed across geographic contexts. The study was not designed to compare responses across survey modes, regions, or countries, and all analyses therefore focus on pooled sample estimates. Participation was anonymous and voluntary. Participants did not receive any compensation. If desired, participants could provide an e-mail address to receive the results of the study.

### Measures

Most measures were single items which are described in the Results section and additionally listed in the Supporting Information (S1 File). Here we describe only the constructed measures.

#### Importance of societal challenges.

Respondents used a scale ranging from 0 (no importance) to 10 (very high importance) to rate the importance of 16 societal challenges*: climate change, air and water pollution, decrease of biodiversity, excessive consumption, violent conflicts/crime, migration, poverty/unemployment, corruption, high taxes, low education, lack of universal healthcare/social security, insufficient food/famine, too rapid population growth, high disease burden, drugs/alcohol, teenage pregnancies.* To simplify the analyses, we conducted a principal components analysis (PCA) with orthogonal varimax rotation and Kaiser normalization. The item *too rapid population growth* had low communality with the other items (0.39; all other items > .63) and was hence excluded. The PCA identified three underlying components, which we interpret as representing: basic welfare (consisting of *low education, poverty/unemployment, violent conflicts/crime, corruption, lack of universal healthcare/social security, migration*; listed in order of the strength of the component loadings), public health and fiscal burden (*teenage pregnancies, drugs/alcohol, high disease burden, high taxes, insufficient food/famine*), and the environment (*air and water pollution, climate change, excessive consumption, decrease of biodiversity*). Further details and the complete results of the PCA are available in the Supporting Information (S2 File). Based on the PCA, we used the average rating of the relevant items as indicators of the importance of basic welfare (6 items; Cronbach’s alpha = .91; 95% Confidence Interval (CI): .89 − .93), public health and fiscal burden (5 items; Cronbach’s alpha = .90; 95% CI: .88 − .93), and the environment (4 items; Cronbach’s alpha = .85; 95% CI: .81 − .89).

#### Willingness to change own behavior.

Respondents used a scale ranging from 0 (disagree) to 10 (strongly agree) to indicate their willingness to change the following behaviors in order to mitigate climate change: *walk rather than take a car for distances under 3 km / 2 miles, recycle as much as possible, avoid unnecessary packaging/plastic bag, reduce water use, switch off lights when not needed, buy local products, cycle/use public transportation instead of car, reduce gas/electricity use at home, take part in political campaign, change my diet, reduce consumption of food/other goods*. We calculated the average of responses to the 11 items as an indicator of respondents’ *willingness to change own behavior* (Cronbach’s alpha = .90; 95% CI: .87 − .92).

#### Age.

Respondents indicated their year of birth, from which we calculated their approximate age in 2018 (the year of data collection).

### Statistical analysis

We used IBM SPSS version 29 to analyze the data. There was very little missing data due to item non-response (6.7% for the item regarding the number of weekly contacts through religious work; ≤ 5.3% missing across all other items). We analyzed the available data without imputing the missing item responses. For measures with nominal response scales or ordinal response scales with fewer than 11 response categories, we calculated the frequency of each response category. We used the median as a measure of central tendency and the interquartile range (IQR) as a measure of dispersion. The IQR represents the spread of the middle 50% of a dataset and is calculated as the difference between the third and first quartiles. For measures with interval response scales and items with ordinal response scales with 11 response categories, we calculated the mean with 95% confidence intervals and standard deviation. Methodological work supports the use of parametric statistics for Likert-type items with 11 response categories [29]. We used paired sample *t-*tests to examine differences in mean importance of addressing the environment relative to addressing basic welfare or public health and fiscal burden. We used Cohen’s *d* as a measure of effect size (0.2 = small, 0.5 = medium, 0.8 = large).

### Inclusivity in global research

Additional information regarding the ethical, cultural, and scientific considerations specific to inclusivity in global research is included in the Supporting Information (S3 Checklist).

## Results

### Existence and importance of climate change

Nearly all respondents agreed that the world’s climate is definitely (73.8%) or probably (24.8%) changing. Before the survey, about half of the respondents had thought about climate change a lot (38.7%) or a great deal (10.7%). About a third had thought about climate change some (36.7%); only a minority had thought about climate change very little (14.0%). Respondents rated the societal importance of environment as rather important, *M =* 7.36, *SD =* 2.00; 0 = no importance to 10 = very high importance. Together, these findings suggest that climate change was both widely recognized and relatively salient among respondents prior to participation in the study.

**Fig 1:**
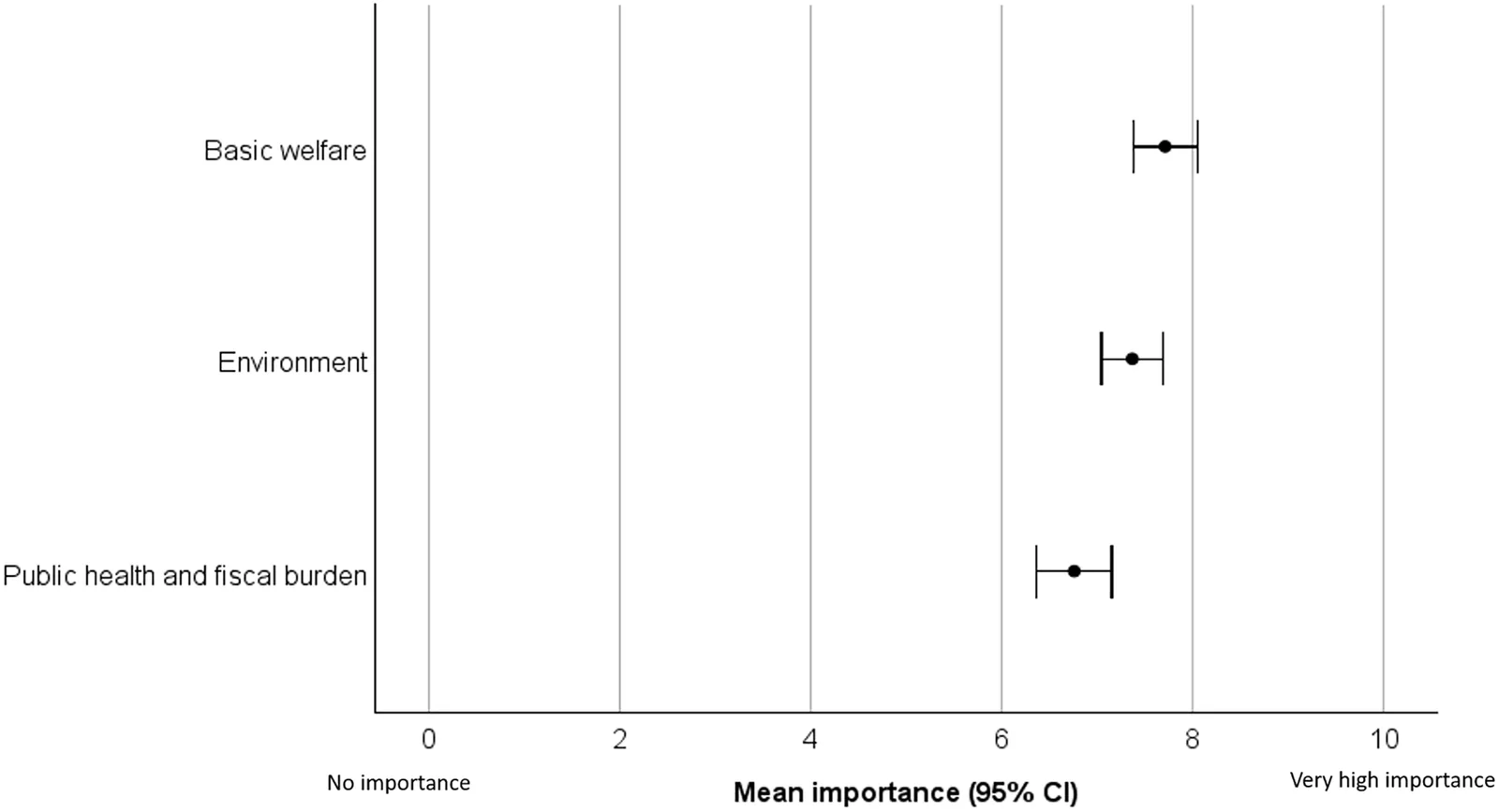
Importance of public health and fiscal burden, the environment, and basic welfare (N = 150 Muslim religious leaders).

Based on the mean ratings, respondents believed that the environment was less important than basic welfare, *t*(148)=2.14, *p =* .03, *d* = .18, but more important than public health and fiscal burden, *t*(148)=−3.39, *p =* .001, *d =* .28 (see Fig 1). This result suggest that respondents are concerned about climate change and viewed environmental issues as important, though not necessarily as the most pressing societal concern.

Most respondents strongly agreed (36.9%) or agreed (38.9%) that climate change should be given priority even if it slows economic growth and some loss of jobs; just a minority disagreed (4.7%) or strongly disagreed (0.7%), while 18.8% neither agreed nor disagreed or had no opinion. Over half of respondents either strongly agreed (26.7%) or agreed (31.3%) that people who use cars and motorbikes should pay higher taxes for polluting the environment; about a quarter either disagreed (19.3%) or strongly disagreed (4.7%), while 18.0% neither agreed nor disagreed or had no opinion. These findings indicate strong support for prioritizing climate action over economic growth in principle, but more mixed views on climate policies that impose direct financial costs on individuals, such as higher taxes on polluting transportation.

### Causes of climate change

**Fig 2:**
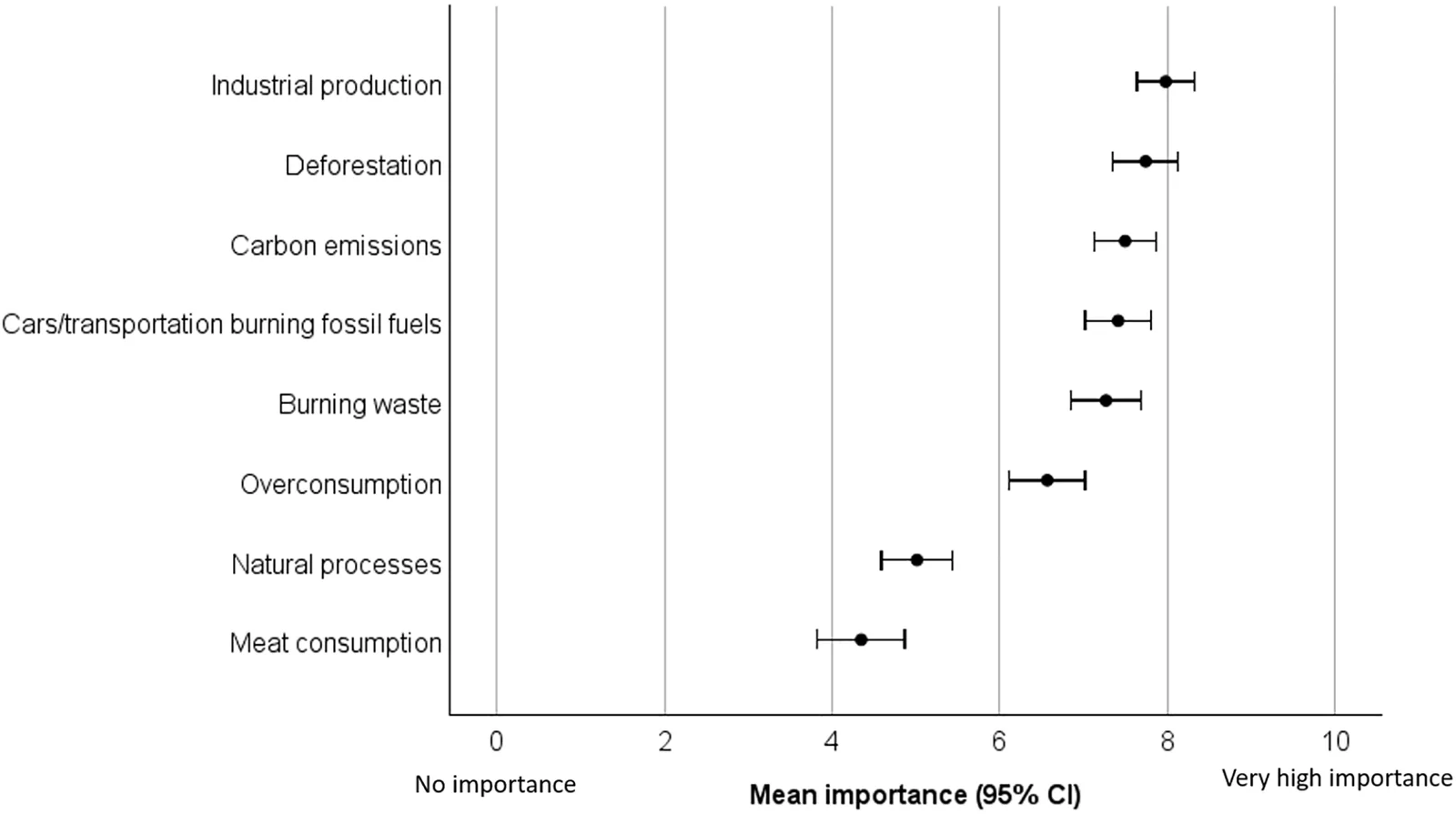
Perceived importance of different causes of climate change (N = 150 Muslim religious leaders).

About half of respondents agreed that climate change was mainly (44.4%) or entirely (7.0%) caused by human activity; 38.7% agreed that natural processes and human activity have contributed to climate change equally. A minority of respondents indicated that climate change is caused mainly (7.0%) or entirely (0.7%) by natural processes. Based on the mean perceived importance, respondents indicated industrial production, deforestation, carbon emissions, cars, and burning waste as the most important causes of climate change (see Fig 2). Overconsumption, and particularly natural processes and meat consumption were perceived as less important causes of climate change. These findings indicate that respondents generally viewed climate change as driven largely by human activity, particularly industrial production and deforestation.

### Consequences of climate change

**Fig 3:**
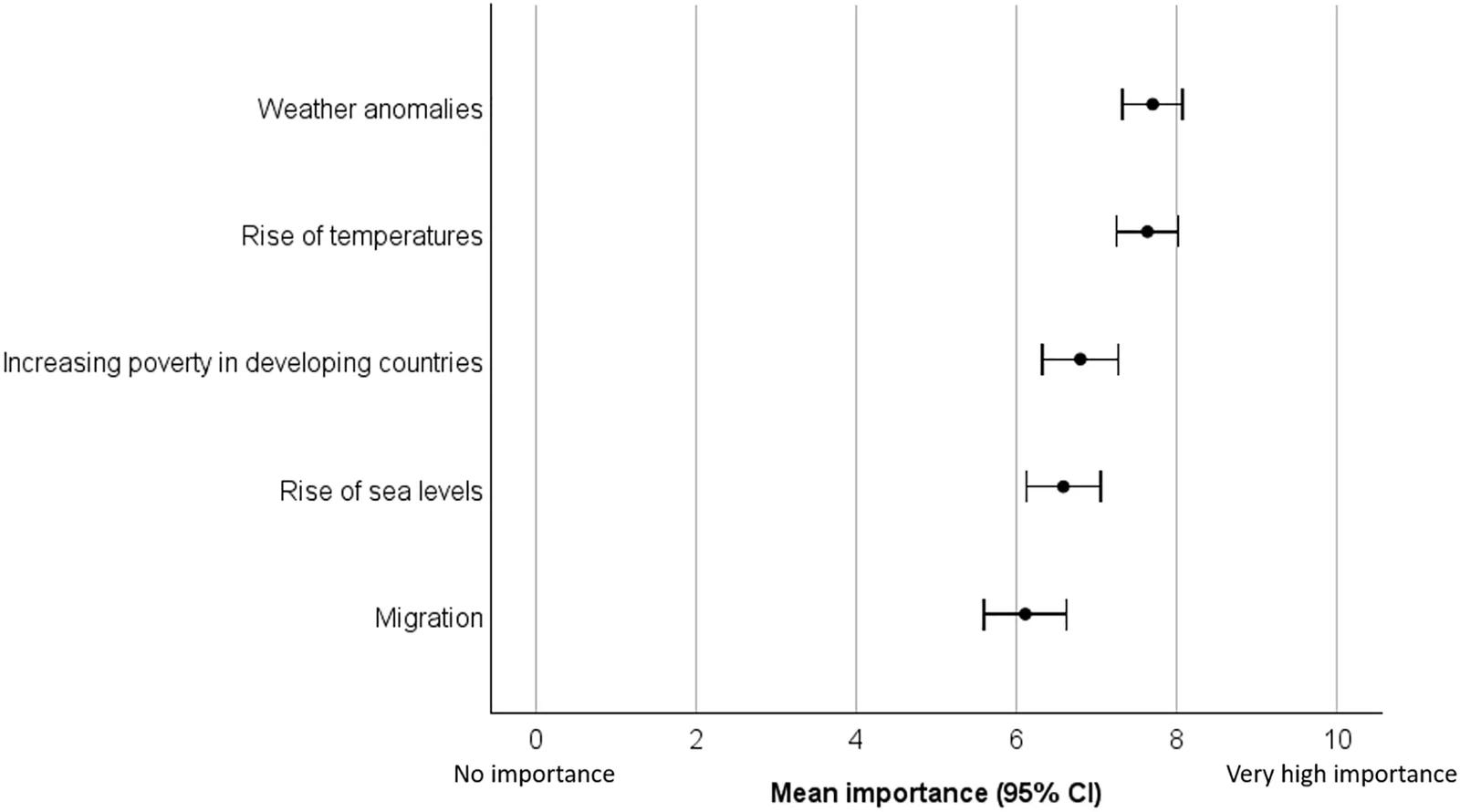
Perceived importance of different consequences of climate change (N = 150 Muslim religious leaders).

Nearly half (48.0%) of respondents believed that climate change was *already* substantially harming people. The remainder believed that climate change will start harming people in 10 years (16.9%), 25 years (14.9%), or 50 years (10.8%). A minority believed that climate change would substantially harm people in 100 years or more (8.8%) or never (0.7%). Based on the mean perceived importance, respondents indicated that weather anomalies such as severe droughts, floods and hurricanes and rising temperatures were the most important consequences of climate change (see Fig 3). Slightly over half of respondents believed that climate change would very likely (16.0%) or possibly (37.3%) lead to the end of human existence. The remainder of the respondents believed that climate change would rather not (13.3%) or surely not (15.3%) lead to the end of human existence; 18.0% indicated no opinion. Together, these findings suggest that most respondents viewed climate change as an acute and serious threat.

### Role of Muslim leaders and other stakeholders

Most respondents (75.3%) agreed that *everyone* is responsible for addressing climate change. A majority also believed that international organizations (68.7%) and world governments (68.0%) were responsible. About half agreed that governments of the highly developed countries (53.3%), international companies (50.0%), local authorities (50.0%), and oil producing countries (43.3%) were responsible. A wide majority strongly agreed (51.0%) or agreed (32.9%) that Muslim religious leaders should be more active in actions related to climate change; only a minority disagreed (2.0%) or strongly disagreed (1.3%), while 10.1% neither agreed nor disagreed and 2.7% had no opinion. Nearly all respondents either strongly agreed (49.0%) or agreed (20.9%) that, as a religious community, Muslims should do more to address the issues of climate change; 7.4% neither agreed nor disagreed, 0.7% strongly disagreed, and 2.0% had no opinion. Most respondents felt a great deal (40.5%), considerable (35.8%), or some (21.6%) responsibility to address climate change themselves; just 2.0% felt no responsibility. These findings indicate that respondents viewed climate action as a shared responsibility and believed that both Muslim communities and Muslim religious leaders should play a more active role. Most respondents (76.2%) indicated that they look toward religious reasoning for guidance on their attitude toward climate change. A wide majority also either strongly agreed (58.4%) or agreed (28.9%) that Islam offers a set of values and principles that help to preserve the Earth and address climate change; 7.4% neither agreed nor disagreed, 0.7% disagreed, and 4.7% indicated no opinion.

In response to an open-ended question asking respondents to identify specific Islamic values, principles, or themes that could inspire environmentally friendly behaviors and help address climate change, respondents referred to *Khalifah* (custodianship; that Muslims/people are the custodians of the Earth appointed by its Creator/Allah; and *Mizan* (balance). Respondents also cited Quran 7:56: “And cause not corruption upon the Earth after its reformation. And invoke Him in fear and aspiration. Indeed, the mercy of Allah is near to the doers of good” as well as Quran 6:28: “There is no creature on [or within] the earth, nor a bird that flies with its wings, except [that they are] communities like you. We have not neglected in the Register a thing. Then unto their Lord they will be gathered.” A number of respondents also stated that people should abstain from overconsumption in line with Quran 7:31: “Eat and drink from the provision of Allah, and do not commit abuse on the earth, spreading corruption.” Some respondents invoked the idea of preserving and caring for water: “And Allah has sent down from the heavens rain, and given life thereby to the earth after its lifelessness. Indeed, in that is a sign for a people who listen” (Quran 16:65). (English translations of Quran verses from Saheeh International.) Together, these findings indicate that respondents rely on religious reasoning when forming climate attitudes and see climate action as consistent with Islamic values and teachings.

### Current actions and future action potential

**Fig 4:**
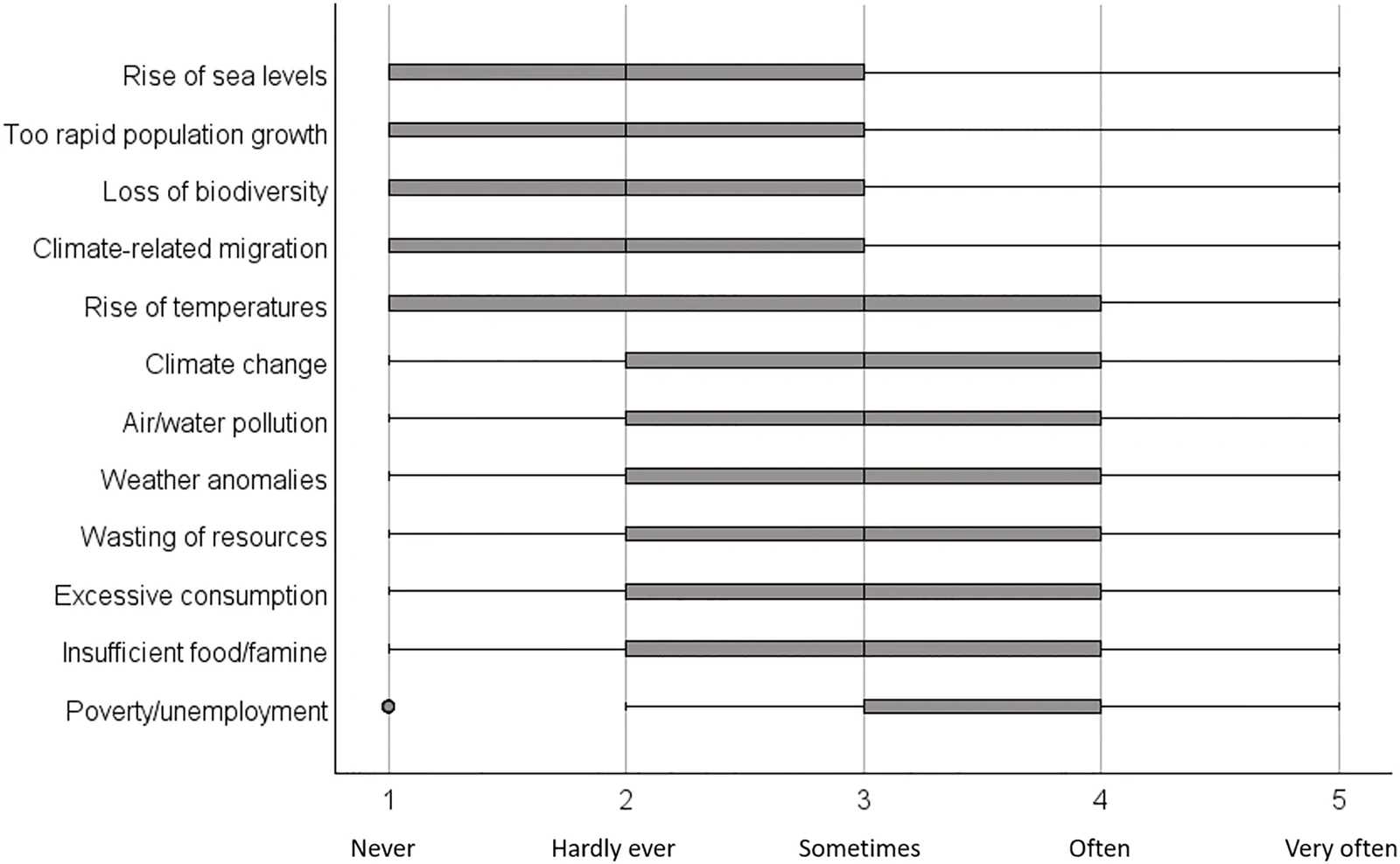
Box plot of self-reported frequency of addressing different topics during preaching, religious work or interaction with congregation in last months (N = 150 Muslim religious leaders).

Respondents were divided on whether leaders of their religious community adequately addressed climate change in communications with their congregation: 16.9% strongly agreed, 27.0% agreed*,* 20.3% disagreed and 11.5% strongly disagreed; the rest neither agreed nor disagreed (16.0%) or had no opinion (8.0%). In their own religious work, respondents had typically addressed climate change and most other environmental topics sometimes during the past months (see Fig 4). Notably, respondents had typically addressed climate-related migration, loss of biodiversity, too rapid population growth, and rise of sea levels less frequently than poverty/unemployment, climate change, and other environmental topics. These findings indicate that respondents had rather mixed perceptions regarding how adequately leaders of their religious community addressed climate change, and typically addressed climate change and environmental issues only occasionally in their own work.

**Fig 5:**
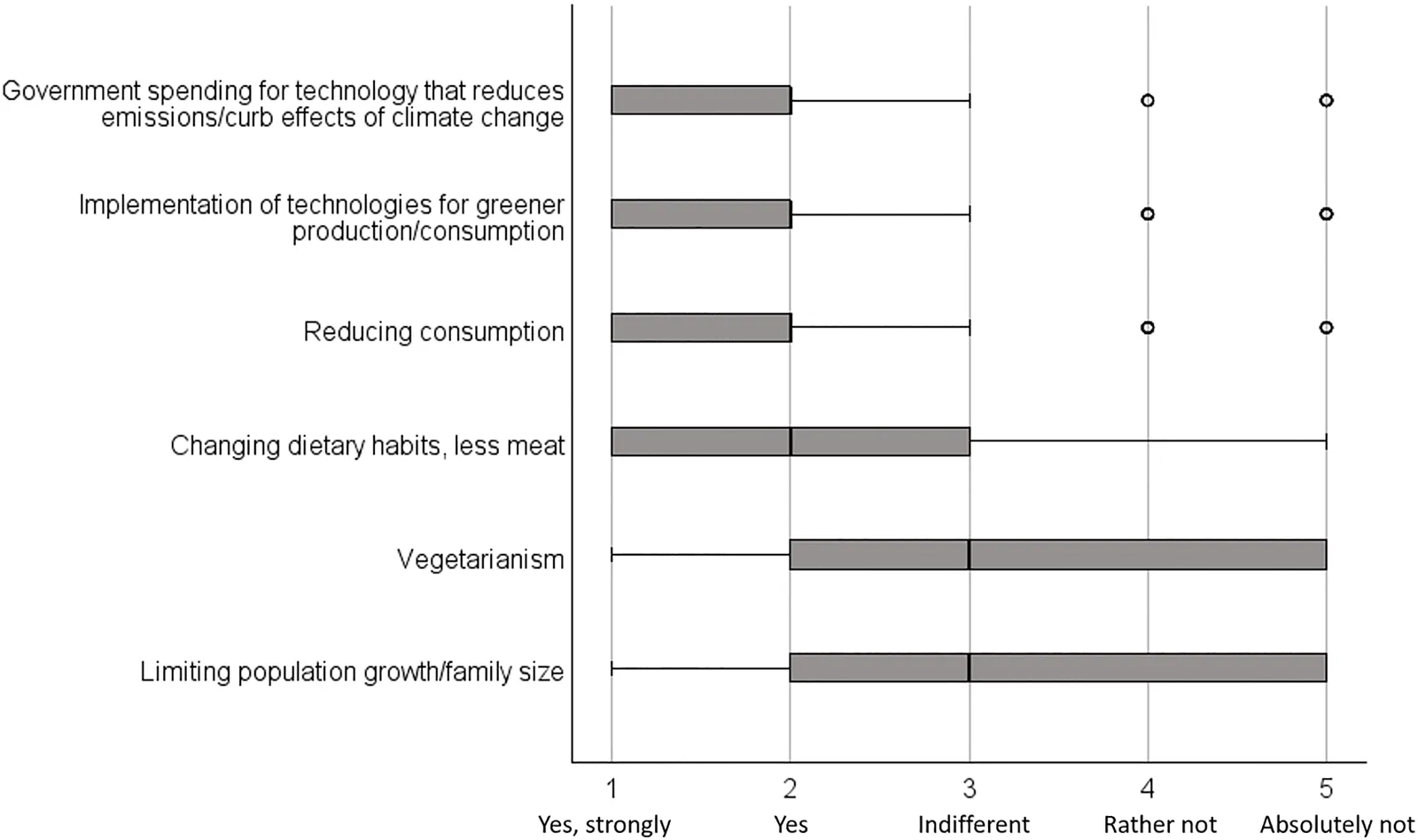
Box plot of respondents’ willingness to promote six climate mitigation actions (N = 150 Muslim leaders). Box plots exclude the minority of respondents (6.8-13.3%) who indicated that they would need more information to decide.

As religious leaders, most respondents were willing to promote government spending on technologies that curb emissions and effects of climate change, the implementation of new technologies to promote greener production and consumption, and reduced consumption (see Fig 5). There was less willingness to support change in dietary habits and less meat consumption; vegetarianism; or limits to population growth and family size relative to other actions. Notably, the range and IQR indicate relatively high heterogeneity in respondents’ willingness to encourage vegetarianism and limit population growth and family size compared with other climate mitigation measures. A minority of respondents (6.8–13.3%) indicated that they would need more information to decide. These findings indicate broad willingness to promote many climate mitigation actions, alongside greater reluctance to promote reduced meat consumption or fertility limitation.

**Fig 6:**
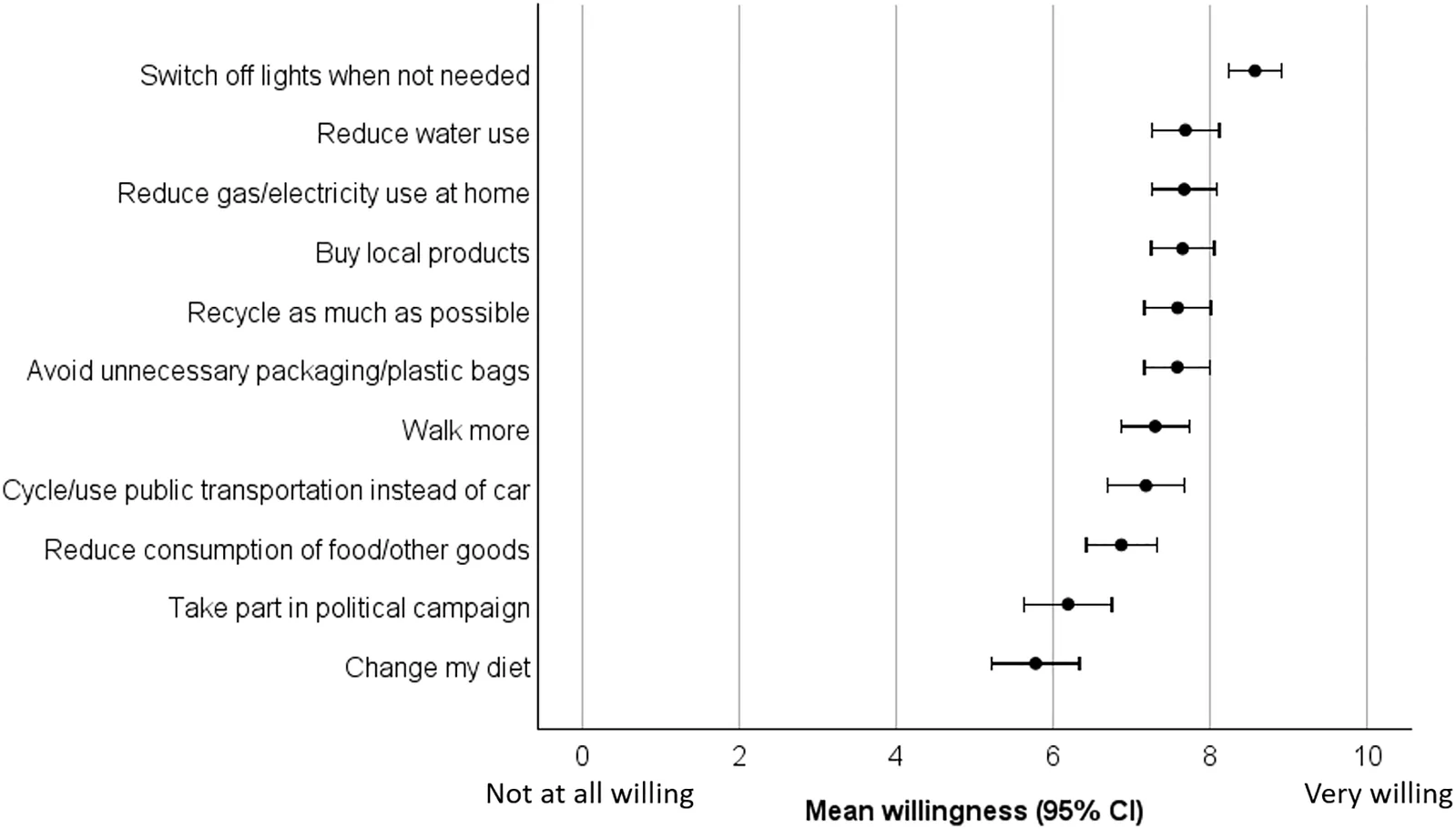
Willingness to personally adopt different climate mitigation behaviors (N = 150 Muslim religious leaders).

Respondents were highly willing to change their own behavior to avoid climate change, *M* = 7.27; SD = 1.95; 0 = not at all willing, 10 = highly willing. Based on the mean willingness to engage in each of 11 different behaviors, they were most willing to switch off the lights when not needed, and least willing to change their diet (see Fig 6). The majority of respondents were either very (28.4%) or quite (48.0%) interested in politics; 14.9% were hardly interested, and 8.8% were not at all interested. Most respondents also thought religion had either a very important (41.2%) or important (37.2%) role to play in politics related to climate change. However, as a group, respondents were only moderately willing to participate in a political campaign themselves, *M* = 6.19, SD = 3.46, 0 = disagree to 10 = strongly agree. Together, these findings suggest that respondents expressed strong willingness to engage in climate-mitigation behaviors and viewed religion as relevant to climate politics, but were comparatively less willing to engage in direct political activism themselves.

## Discussion

So far, relatively little is known about what religious leaders of different faiths think about climate change, and the existing research has been predominantly qualitative, narrow in scope, and/or focused on Christian leaders. This is an important gap because religious leaders have significant potential for spreading knowledge about climate change and motivating environmentally sustainable behavior. Caring for the natural world is a principle deeply embedded in all of the world’s five major religions (Christianity, Judaism, Islam, Hinduism, and Buddhism) [30,31]. Religious organizations and leaders have been increasingly involved in national and international meetings on the environment, and there has been substantial growth in scholarly articles on ecology and religion [32]. Relative to other public figures, religious leaders may be especially well positioned to influence the climate views of conservative groups. In the Middle East, for instance, Muslims who self-identify as religious and support Islamist government are relatively unconcerned about climate change; moreover, the climate change movement is associated with Western-inspired pro-democracy movements that are popularly interpreted as anti-state and anti-Islam [33]. Muslim religious leaders may therefore be especially effective in engaging such religiously conservative groups and shaping their views on climate change.

Based on a global convenience sample, our results offer exploratory insights on a wide range of Muslim religious leaders’ beliefs and attitudes about climate change as well as their willingness to personally adopt and promote different environmentally sustainable behaviors. Overall, our results suggest that Muslim leaders are highly aware of climate change and take its consequences seriously. Nearly all of our respondents agreed that the climate is driven, at least in part, by human activity. Most respondents believed that climate change is already substantially harming people or will begin to do so within the next 10 years, and slightly over half believed that climate change could ultimately threaten human existence.

Nevertheless, respondents had not been regularly addressing climate change in their religious work. This result is consistent with Syropoulos and Sparkman [14], who found that nearly all U.S. religious leaders surveyed – most of whom were Christian – believed in anthropogenic climate change to some degree, yet only a minority had addressed climate change with their congregation more than once or twice. The observation that both Muslim leaders in the current study and primarily Christian leaders in the study from Syropoulos and Sparkman [14] acknowledge anthropogenic climate change yet rarely address it in their religious work suggests convergence in behavior, but not necessarily the underlying causes. Some reasons may cut across faith groups, such as deprioritizing climate change in the face of more immediate concerns (providing moral or spiritual guidance; supporting community members during crises), or having limited guidance on how to meaningfully integrate environmental issues into their religious work. Other reasons may be specific to particular faiths or social contexts. Future research should examine why religious leaders do not engage with climate change more actively, recognizing that the reasons may vary across theological traditions, institutional structures, political contexts, and geographies.

Although our findings are based on a convenience sample and should be interpreted cautiously, our results also suggest that there may be potential for Muslim religious leaders to take more action to address climate change. Most respondents agreed that Islam offers values and principles that help to preserve the Earth and address climate change. This finding echoes the work of Muslim environmentalists, who cite *Khalifa*, *Mizan*, *Tawhid* and *Maslahah* as principles guiding Muslims’ relationship with the environment [34–36]. As noted earlier, *Khalifa* embodies the notion of humans as stewards of God’s creation, similar to the notion of stewardship in Christianity and Judaism, while *Mizan* embodies the notion of balance and harmony between all parts of creation. *Tawhid* refers to oneness and the unity of creation including humans and nature. Finally, *Maslahah* refers to prioritizing public over private interest and caring for future generations.

In addition to viewing climate action as consistent with Islamic values and principles, the vast majority of respondents felt at least some personal responsibility for addressing climate change, and agreed that their religious community should do more to address the issue. They expressed high willingness to personally adopt climate mitigation behaviors, and were also willing to promote government spending on technologies, the implementation of technologies, and reduced consumption through their religious work. Given their relatively large networks of influence (e.g., about half of respondents had weekly contact with 100 or more people, see Table 1), these finding suggest that Muslim religious leaders may be well positioned to spread awareness and encourage (some) climate mitigation behaviors among a large number of people.

Our results are broadly consistent with the increasing number of Muslim groups and initiatives that aim to tackle climate change and advance sustainable development, including Global Muslim Climate Network, The Islamic Foundation for Ecology and Environmental Sciences, Two Billion Strong, and Faithfully Sustainable. Curiously, despite expressing interest in politics and believing that religion has an important role to play in the politics of climate change, the religious leaders in our sample were somewhat reluctant to participate in political campaigns themselves. Future research should examine whether interventions that strengthen the political self-efficacy of Muslim religious leaders could increase their engagement in climate-related political action.

Wynes and Nicholas [37] suggested that having one fewer child is the most effective way that individuals can mitigate climate change, at least in developed countries where consumption is high. Our results, however, suggest that the Muslim religious leaders in our sample were reluctant to promote limiting fertility as a means for mitigating climate change. This reluctance is notable given that many Muslim scholars consider fertility regulation permissible within Islam [38,39], and prior research in Jordan found that Muslim religious leaders did not express a preference for large families and were generally supportive of family planning [40]. Although our data do not allow us to identify the reasons underlying respondents’ reluctance, several explanations are possible. Respondents may have had doubts about whether Islam considers climate concerns to be an acceptable justification for limiting fertility, or doubts about the effectiveness of limiting fertility as a means of mitigating climate change and/or practical and ethical concerns. Such doubts and concerns are not unique to Muslim communities [e.g., 41–43].

Another highly impactful way individuals can reduce their environmental impact is to reduce meat consumption [37,44]. However, our results suggest that the Muslim religious leaders in our sample are hesitant to promote vegetarianism as a climate mitigation strategy. This finding is notable given that vegetarianism is relatively uncommon among Muslims compared with some other religious groups. In India, for instance, the vast majority of Jains say they are vegetarian (92%), compared with 44% of Hindus, and just 8% of Muslims and 10% of Christians [45]. While vegetarianism is generally considered permissible within Islam, some dissenting views exist [46,47]. Future research should examine how Muslim religious leaders perceive vegetarianism and reduced meat consumption as forms of climate action, and whether reduced meat consumption framed in terms of moderation or concern for animal welfare rooted in Islamic ethical principles may be viewed more favorably [48].

### Strengths, limitations, and future research

Drawing on a global convenience sample, our study provides initial empirical insights into Muslim leaders’ beliefs about climate change, its causes, and potential responses. Existing surveys of religious leaders’ climate views have been limited in scope and have focused primarily on Christian leaders. Much of the existing literature on Islam, the environment, and climate change is theoretical and centered on Islamic environmental ethics [for a literature review, see 34]. Our study extends this literature by providing quantitative survey data on the climate views of Muslim religious leaders. It also complements broader research examining how religion, both as a belief system and as a social identity, is related to people’s views of climate change and their environmental behavior [for reviews, see 18,49].

Our results are based on a diverse convenience sample of Muslim leaders from 29 countries in the Global North and South, including Muslim leaders in a number of countries where data collection is difficult due to ongoing conflicts. We caution, however, that our results may not be representative of Muslim leaders worldwide. There is no comprehensive or standardized global registry of individuals who qualify as Muslim religious leaders, making it impossible to construct a complete sampling frame from which to draw a random sample or systematically assess the magnitude of selection bias.

Although our sampling strategy enabled us to efficiently recruit a diverse and global sample of Muslim religious leaders, it may also limit the generalizability of our findings. Snowball sampling can introduce bias, as people often associate with others who share similar views. In addition, the Muslim leaders in our study who voluntarily chose to take part in a survey on climate change beliefs may have been more interested in the topic than Muslim religious leaders more broadly. Our results may therefore overestimate climate change awareness, concern, and willingness to adopt and promote climate mitigation behaviors among Muslim religious leaders. Because our findings are based on a heterogeneous convenience sample whose representativeness cannot be assessed, we are unable to make actionable policy recommendations. Moreover, our sample size limited our ability to investigate differences among Muslim leaders (e.g., differences associated with country, school of jurisprudence, age).

Other limitations concern the survey instrument and data collection procedures. No back-translation or formal validation procedures were conducted for the translated survey instruments, which means that we cannot ensure cross-linguistic equivalence across all survey versions. Although we do not compare responses across language groups, subtle differences in wording across language versions may have introduced measurement error or systematic differences in how respondents interpreted survey items, which could affect pooled estimates across the full sample. We also collected data using both offline and online questionnaires. Online and paper questionnaires share several key characteristics (e.g., they are both self-administered and visually mediated), and empirical research suggests they often yield comparable results [50]. However, survey responses do sometimes vary depending on whether a questionnaire is completed online or offline [51]. Because survey mode was not randomly assigned and was unevenly distributed across geographic contexts, our data do not allow us to disentangle mode effects from geographical variation. The resulting confounding of survey mode and geographic region prevents meaningful descriptive or inferential comparisons between online and paper respondents. For transparency, descriptive statistics for six study constructs by survey mode are provided in the Supporting Information (S4 File).

Future studies should replicate and build on our results by recruiting larger samples of Muslim religious leaders, comparing their climate views with those of religious leaders from other faith groups (e.g., assessing whether reluctance to encourage vegetarianism or smaller families for environmental reasons is particular to Muslim leaders), investigating potential survey mode effects, and exploring Muslim religious leaders’ attitudes toward other high-impact climate mitigation behaviors not included in the current study, such as reducing food waste, flying less, or switching to renewable energy sources [37,44]. Research that combines empirical approaches with insights from scholarship on Islamic ethics could help clarify why Muslim religious leaders appear to be reluctant to promote dietary change and fertility limitation as strategies for mitigating climate change.

## Conclusion

This study provides exploratory insights into the climate change beliefs of a global convenience sample of Muslim religious leaders. Our findings suggest that many respondents recognize climate change as human-caused, believe it poses an acute threat, view climate action as consistent with Islamic values, and express willingness to support certain climate mitigation behaviors. Respondents’ reluctance to encourage reduced meat consumption and fertility limitation as climate mitigation strategies warrants future research. Given the study’s methodological limitations, these findings should be interpreted cautiously. Nevertheless, this study offers an initial empirical foundation for hypothesis generation and future research on the role of religious leaders in shaping climate attitudes and advancing climate action.

## Supporting information

S1 File Questionnaire items and descriptive statistics. (DOCX)

S2 File Details and results of the principal components analysis of the importance of different societal challenges. (DOCX)

S3 Checklist Inclusivity in global research questionnaire. (DOCX)

S4 File Descriptive statistics for selected study variables by survey mode. (DOCX)

## References

[1] Pew Research Center. The changing global religious landscape. Washington (DC). 2017.

[2] Cavdar D, Tepe B, Saribay SA, Yilmaz O. Moral framing effects on environmental attitudes: a conceptual replication and extension of Feinberg and Willer (2013). J Environ Psychol. 2025;108:102827. doi: 10.1016/j.jenvp.2025.102827

[3] Feinberg M, Willer R. The moral roots of environmental attitudes. Psychological Science. 2013;24(1):56–62. doi: 10.1177/0956797612449177 23228937

[4] Stern PC, Dietz T, Abel T, Guagnano GA, Kalof L. A value-belief-norm theory of support for social movements: the case of environmentalism. Human Ecology Review. 1999;6(2):81–97.

[5] Masson T, Fritsche I. We need climate change mitigation and climate change mitigation needs the ‘We’: a state-of-the-art review of social identity effects motivating climate change action. Current Opinion in Behavioral Sciences. 2021;42:89–96. doi: 10.1016/j.cobeha.2021.04.006

[6] Posas P. Roles of religion and ethics in addressing climate change. Ethics Sci Environ Polit. 2007;9:31–49. doi: 10.3354/esep007031

[7] Hornsey MJ, Harris EA, Bain PG, Fielding KS. Meta-analyses of the determinants and outcomes of belief in climate change. Nature Clim Change. 2016;6(6):622–6. doi: 10.1038/nclimate2943

[8] Caprara GV, Vecchione M, Schwartz SH, Schoen H, Bain PG, Silvester J, et al. The contribution of religiosity to ideology: empirical evidences from five continents. Cross-Cultural Research. 2018;52(5):524–41. doi: 10.1177/1069397118774233

[9] Freiling I, Cacciatore MA, McKasy M. Religious values and confidence in science: perceived tensions and common ground. PLoS One. 2025;20(9):e0332477. doi: 10.1371/journal.pone.0332477 40971850 PMC12448960

[10] Van Boven L, Sherman DK. Elite influence on public attitudes about climate policy. Current Opinion in Behavioral Sciences. 2021;42:83–8. doi: 10.1016/j.cobeha.2021.03.023

[11] Brulle RJ, Carmichael J, Jenkins JC. Shifting public opinion on climate change: an empirical assessment of factors influencing concern over climate change in the U.S., 2002–2010. Climatic Change. 2012;114(2):169–88. doi: 10.1007/s10584-012-0403-y

[12] Shin F, Preston JL. Green as the gospel: the power of stewardship messages to improve climate change attitudes. Psychology of Religion and Spirituality. 2021;13(4):437–47. doi: 10.1037/rel0000249

[13] Buckley DT. Religious elite cues, internal division, and the impact of Pope Francis’ Laudato Si’. Politics and Religion. 2020;15(1):1–33. doi: 10.1017/s175504832000067x

[14] Syropoulos S, Sparkman G. Most Christian American religious leaders silently believe in climate change, and informing their congregation can help open dialogue. Proc Natl Acad Sci U S A. 2025;122(13):e2419705122. doi: 10.1073/pnas.2419705122 40131947 PMC12002303

[15] Myers TA, Roser-Renouf C, Maibach E, Leiserowitz A. Exposure to the Pope’s climate change message activated convinced americans to take certain activism actions. Glob Chall. 2017;1(4):1600019. doi: 10.1002/gch2.201600019 31565270 PMC6607370

[16] Pope F. Laudate deum: apostolic exhortation to all people of good will on the climate crisis. 2023.

[17] Dalai Lama Tenzing G, Hanh ZMTN, Karmapa G, Mahathero HHDD, Murayama RH, Sunim HEJ, et al. Buddhist climate change statement to world leaders. 2015.

[18] Jenkins W, Berry E, Kreider LB. Religion and climate change. Annual Review of Environ Resources. 2018;43(1):85–108. doi: 10.1146/annurev-environ-102017-025855

[19] Wiktor-Mach D, Pędziwiatr K. Polish Catholic environmentalism as the counterculture movement. Environmental Politics. 2024;33(6):976–96. doi: 10.1080/09644016.2024.2313656

[20] Wilkins D. Catholic clerical responses to climate change and Pope Francis’s Laudato Si’. Environment and Planning E: Nature and Space. 2020;5(1):146–68. doi: 10.1177/2514848620974029

[21] Djupe PA, Gilbert CP. The political voice of clergy. J Politics. 2002;64(2):596–609. doi: 10.1111/1468-2508.00142

[22] Guth JL, Green JC, Kellstedt LA, Smidt CE. Faith and the environment: religious beliefs and attitudes on environmental policy. American J Political Science. 1995;39(2):364. doi: 10.2307/2111617

[23] Lifeway Research. Pastors’ views on global warming: Survey of American Protestant pastors. Lifeway Research; 2020. https://lwresearch21.wpengine.com/wp-content/uploads/2020/04/Pastor-Views-on-Global-Warming-2019.pdf

[24] Öhlmann P, Sonntag EA. The religious leaders’ perspectives on corona survey: methods and key results. Religions. 2024;15(12):1474. doi: 10.3390/rel15121474

[25] Notre Dame Global Adaptation Initiative. World wide ranking by vulnerability. 2022.

[26] Namdar R, Karami E, Keshavarz M. Climate change and vulnerability: the case of MENA Countries. IJGI. 2021;10(11):794. doi: 10.3390/ijgi10110794

[27] Pendrill F, Persson UM, Godar J, Kastner T, Moran D, Schmidt S. Share of tropical deforestation. Global Environmental Change. 2019. 1–10. doi: 10.1016/j.gloenvcha.2019.03.002

[28] Skirbekk V, Pedziwiatr K. Sustainability and climate change in major religions with a focus on Islam. Birmingham: Humanitarian Academy for Development; 2018.

[29] Wu H, Leung S-O. Can likert scales be treated as interval scales?—A simulation study. J Social Service Research. 2017;43(4):527–32. doi: 10.1080/01488376.2017.1329775

[30] Agusalim L, Karim M. Religiosity and climate change: An eco-religious approach. Environmental & Socio-economic Studies. 2024;12(1):35–50. doi: 10.2478/environ-2024-0004

[31] Marshall G, Corner A, Roberts O, Clarke J. Faith and climate change - a guide to talking with the five major faiths. Oxford: Climate Outreach; 2016.

[32] Hitzhusen GE, Tucker ME. The potential of religion for Earth Stewardship. Frontiers in Ecol Environ. 2013;11(7):368–76. doi: 10.1890/120322

[33] Mazaheri N. Faith in science: religion and climate change attitudes in the Middle East. Global Environmental Politics. 2024;24(1):52–75. doi: 10.1162/glep_a_00720

[34] Koehrsen J. Muslims and climate change: How Islam, Muslim organizations, and religious leaders influence climate change perceptions and mitigation activities. WIREs Climate Change. 2021;12(3). doi: 10.1002/wcc.702

[35] Islamic Relief Worldwide. International Islamic climate change symposium: summary. Islamic Relief Worldwide. 2015.

[36] Abdelzaher DM, Abdelzaher A. Beyond environmental regulations: exploring the potential of “eco-islam” in boosting environmental ethics within SMEs in Arab Markets. J Bus Ethics. 2015;145(2):357–71. doi: 10.1007/s10551-015-2833-8

[37] Wynes S, Nicholas KA. The climate mitigation gap: education and government recommendations miss the most effective individual actions. Environ Res Lett. 2017;12(7):074024. doi: 10.1088/1748-9326/aa7541

[38] Oraby D. Islam and contraception: a missed opportunity. Repro Female Child Health. 2023;3(1). doi: 10.1002/rfc2.49

[39] Roudi-Fahimi F. Islam and family planning. Washington, D.C.: Population Reference Bureau; 2004.

[40] Underwood C. Islamic precepts and family planning: the perceptions of jordanian religious leaders and their constituents. Int Family Planning Perspectives. 2000;26(3):110. doi: 10.2307/2648299

[41] Sierra Club. Population policy. 2022.

[42] Cafaro P. Climate ethics and population policy: a review of recent philosophical work. WIREs Climate Change. 2021;13(2). doi: 10.1002/wcc.748

[43] Budolfson M, Spears D. Population ethics and the prospects for fertility policy as climate mitigation policy. J Dev Stud. 2021;57(9):1499–510. doi: 10.1080/00220388.2021.1915481 34421127 PMC8373053

[44] Ivanova D, Barrett J, Wiedenhofer D, Macura B, Callaghan M, Creutzig F. Quantifying the potential for climate change mitigation of consumption options. Environ Res Lett. 2020;15(9):093001. doi: 10.1088/1748-9326/ab8589

[45] Corichi M. Eight-in-ten Indians limit meat in their diets, and four-in-ten consider themselves vegetarian. Washington, D.C.: Pew Research Center; 2021.

[46] Ali K. Muslims and meat-eating. J Religious Ethics. 2015;43(2):268–88. doi: 10.1111/jore.12097

[47] Islam MN, Islam MS. Human-animal relationship: understanding animal rights in the Islamic ecological paradigm. J Study of Religions and Ideologies. 2015;14(41):96–126.

[48] Mukhtar M, Todd M-J. An Islamic framework for animal ethics: Widening the conversation to include Islamic ethical vegetarianism. Critical Res Religion. 2023;11(3):263–80. doi: 10.1177/20503032231174209

[49] Sachdeva S. Religious identity, beliefs, and views about climate change. In: von Storch H, editor. Oxford Research Encyclopedia of Climate Science. New York: Oxford Academic; 2016. doi: 10.1093/acrefore/9780190228620.013.335

[50] DeLeeuw ED. Mixed-mode: past, present, and future. Survey Res Methods. 2018;12(2):75–89. doi: 10.18148/srm/2018.v12i2.7402

[51] Hox J, de Leeuw E, Klausch T. Mixed-Mode Research. In: Biemer P, de Leeuw E, Eckman S, Edwards B, Kreuter F, Lyberg L, editors. Total survey error in practice. Hoboken (NJ): John Wiley & Sons; 2017. 511–30. doi: 10.1002/9781119041702.ch23

