# Supplementary material for: Muslim religious leaders’ views of climate change: An international survey
Source: PLoS One. 2026 Aug 25;21(8):e0353201. doi: 10.1371/journal.pone.0353201 (PMC13505925; doi:10.1371/journal.pone.0353201)
Supplement: Supplementary file 1 [file pone.0353201.s009.pdf]

Dear Editor,

Thank you for your detailed feedback. We have carefully revised the manuscript in response to all remaining concerns and provide a detailed point-by-point response in the attached document.

We appreciate your time and consideration.

Sincerely,

Dr. Catherine E. Bowen

Also on behalf of the other authors
