# Supplementary material for: Muslim religious leaders’ views of climate change: An international survey
Source: PLoS One. 2026 Aug 25;21(8):e0353201. doi: 10.1371/journal.pone.0353201 (PMC13505925; doi:10.1371/journal.pone.0353201)
Supplement: Supplementary file 2 [file pone.0353201.s001.docx]

**S1 file. Questionnaire items and descriptive statistics.**

*Do you think the worlds climate is changing?*

|  |  | N | % | Valid % |
| --- | --- | --- | --- | --- |
|  | Definitely changing | 110 | 73.3% | 73.8% |
|  | Probably changing | 37 | 24.7% | 24.8% |
|  | Probably not changing | 1 | 0.7% | 0.7% |
|  | I don't have opinion on this | 1 | 0.7% | 0.7% |
|  | No response | 1 | 0.7% |  |

*Do you think climate change is caused by natural processes, human activity, or both*?

|  |  | | N | % | Valid % |
| --- | --- | --- | --- | --- | --- |
|  | Entirely natural process | | 1 | 0.7% | 0.7% |
|  | Mainly natural process | | 8 | 5.3% | 5.6% |
|  | About equally natural process and human activity | | 55 | 36.7% | 38.7% |
|  | Mainly human activity | | 63 | 42.0% | 44.4% |
|  | Entirely human activity | | 10 | 6.7% | 7.0% |
|  | I don't have an opinion on this | | 5 | 3.3% | 3.5% |
|  | No response |  | 8 | 5.3% |  |

*How much have you thought about climate change before today?*

|  |  | N | % |
| --- | --- | --- | --- |
|  | Very little | 21 | 14.0% |
|  | Some | 55 | 36.7% |
|  | A lot | 58 | 38.7% |
|  | A great deal | 16 | 10.7% |

*What do you think causes climate change? (0 – No importance and 10 – Very high importance)*

|  |  | N | Min. | Max. | M | SD |
| --- | --- | --- | --- | --- | --- | --- |
|  | Carbon emissions | 149 | 0 | 10 | 7.50 | 2.31 |
|  | Cars and other means of transportation burning fossil fuels | 150 | 0 | 10 | 7.41 | 2.46 |
|  | Meat consumption | 149 | 0 | 10 | 4.35 | 3.22 |
|  | Industrial production | 150 | 1 | 10 | 7.98 | 2.12 |
|  | Deforestation | 147 | 0 | 10 | 7.74 | 2.38 |
|  | Burning waste | 149 | 0 | 10 | 7.27 | 2.55 |
|  | Overconsumption | 148 | 0 | 10 | 6.57 | 2.79 |
|  | Natural processes | 147 | 0 | 10 | 5.01 | 2.61 |

*What in your view are the most troubling effects of climate change? (0 – No importance and 10 – Very high importance)*

|  |  | N | Min. | Max. | M | SD |
| --- | --- | --- | --- | --- | --- | --- |
|  | Rise of temperatures | 149 | 0 | 10 | 7.63 | 2.39 |
|  | Rise of sea levels | 146 | 0 | 10 | 6.58 | 2.83 |
|  | Weather anomalies (severe droughts, floods, more frequent hurricanes) | 148 | 0 | 10 | 7.70 | 2.31 |
|  | Growing poverty in developing countries | 146 | 0 | 10 | 6.79 | 2.89 |
|  | Migrations of people | 149 | 0 | 10 | 6.11 | 3.17 |

*When will climate change harm people substantially?*

|  | | N | % | Valid % |
| --- | --- | --- | --- | --- |
|  | Already now | 71 | 47.3% | 48.3% |
|  | In 10 years | 25 | 16.7% | 17.0% |
|  | In 25 years | 22 | 14.7% | 15.0% |
|  | In 50 years | 16 | 10.7% | 10.9% |
|  | In 100 years or more | 13 | 8.7% | 8.8% |
|  | Never | 1 | 0.7% | 0.7% |
|  | No response | 2 | 1.3% |  |

*How likely it is that the climate changes will lead to the end of human existence in the world?*

|  | N | % |
| --- | --- | --- |
| Very likely | 24 | 16.0% |
| Possibly yes | 56 | 37.3% |
| Rather not | 20 | 13.3% |
| Surely not | 23 | 15.3% |
| I don't know | 27 | 18.0% |

*Do you agree that dealing with the problem of climate change should be given priority, even if it causes slower economic growth and some loss of jobs?*

|  |  | N | % | Valid % |
| --- | --- | --- | --- | --- |
|  | Strongly agree | 55 | 36.7% | 36.9% |
|  | Agree | 58 | 38.7% | 38.9% |
|  | Neither agree nor disagree | 21 | 14.0% | 14.1% |
|  | Disagree | 7 | 4.7% | 4.7% |
|  | Strongly disagree | 1 | 0.7% | 0.7% |
|  | I have no opinion | 7 | 4.7% | 4.7% |
|  | No response | 1 | 0.7% |  |

*Users of cars/motorbikes should pay higher taxes for polluting the environment.*

|  | N | % |
| --- | --- | --- |
| Strongly agree | 40 | 26.7% |
| Agree | 47 | 31.3% |
| Neither agree nor disagree | 24 | 16.0% |
| Disagree | 29 | 19.3% |
| Strongly agree | 7 | 4.7% |
| I have no opinion | 3 | 2.0% |

*How important are the following societal challenges? (0 – No importance and 10 – Very high importance)*

|  |  | N | Min. | Max. | *M* | *SD* |
| --- | --- | --- | --- | --- | --- | --- |
|  | Climate change | 149 | 0 | 10 | 7.45 | 2.44 |
|  | Air and water pollution | 149 | 0 | 10 | 8.02 | 2.05 |
|  | Decrease of biodiversity and that many species (both animals and plants) go extinct | 147 | 0 | 10 | 7.10 | 2.65 |
|  | Excessive consumption | 148 | 0 | 10 | 6.89 | 2.37 |
|  | Violent conflicts and crime | 146 | 0 | 10 | 7.97 | 2.42 |
|  | Migrations of people | 146 | 0 | 10 | 6.84 | 2.73 |
|  | Poverty and unemployment | 147 | 0 | 10 | 7.56 | 2.49 |
|  | Corruption | 148 | 0 | 10 | 8.10 | 2.34 |
|  | High taxes | 147 | 0 | 10 | 6.54 | 2.79 |
|  | Low education | 146 | 0 | 10 | 7.93 | 2.41 |
|  | Lack of universal healthcare and social security | 148 | 1 | 10 | 7.92 | 2.25 |
|  | Insufficient food and famine | 146 | 0 | 10 | 7.40 | 2.70 |
|  | Importance: rapid population growth | 145 | 0 | 10 | 6.08 | 3.05 |
|  | A high disease burden | 149 | 0 | 10 | 6.94 | 2.56 |
|  | Too much drugs and alcohol use | 148 | 0 | 10 | 7.10 | 2.85 |
|  | Teenage pregnancies | 148 | 0 | 10 | 5.89 | 3.26 |

*Whose responsibility it is to address the problem of the climate change? (multiple answers possible)*

|  |  | N | % |
| --- | --- | --- | --- |
|  | International organizations (e.g., UN, EC) | 103 | 68.7% |
|  | Governments of the highly developed countries | 80 | 53.3% |
|  | Oil producing countries | 65 | 43.3% |
|  | Government of every country including mine | 102 | 68.0% |
|  | International companies | 75 | 50.0% |
|  | Local authorities | 75 | 50.0% |
|  | Every person on Earth | 113 | 75.3% |

*The leaders of my religious community adequately address climate change in their communications with their congregations.*

|  |  | N | % | Valid % |
| --- | --- | --- | --- | --- |
|  | Strongly agree | 25 | 16.7% | 16.9% |
|  | Agree | 40 | 26.7% | 27.0% |
|  | Neither agree nor disagree | 24 | 16.0% | 16.2% |
|  | Disagree | 30 | 20.0% | 20.3% |
|  | Strongly disagree | 17 | 11.3% | 11.5% |
|  | I have no opinion | 12 | 8.0% | 8.1% |
|  | No response | 2 | 1.3% |  |

*Muslim religious leaders should be more active in actions related to climate change.*

|  |  | N | % | Valid % |
| --- | --- | --- | --- | --- |
|  | Strongly agree | 76 | 50.7% | 51.0% |
|  | Agree | 49 | 32.7% | 32.9% |
|  | Neither agree nor disagree | 15 | 10.0% | 10.1% |
|  | Disagree | 3 | 2.0% | 2.0% |
|  | Strongly disagree | 2 | 1.3% | 1.3% |
|  | I have no opinion | 4 | 2.7% | 2.7% |
|  | No response | 1 | 0.7% |  |

*Islam offers set of values/principles that help preserve the Earth and address climate change.*

|  |  | N | % | Valid % |
| --- | --- | --- | --- | --- |
|  | Strongly agree | 87 | 58.0% | 58.4% |
|  | Agree | 43 | 28.7% | 28.9% |
|  | Neither agree nor disagree | 11 | 7.3% | 7.4% |
|  | Disagree | 1 | 0.7% | 0.7% |
|  | I have no opinion | 7 | 4.7% | 4.7% |
|  | No response | 1 | 0.7% |  |

*What Islamic values or principles or themes are the most important ones to inspire or guide behaviours of Muslims that would be more environmentally friendly and could help to address the issues of climate change. (Please list up to 5)*

(open answer format)

*Would you look for religious reasoning on what your attitude should be toward climate change?*

|  |  | N | % | Valid % |
| --- | --- | --- | --- | --- |
|  | Yes | 112 | 74.7% | 76.2% |
|  | No | 35 | 23.3% | 23.8% |
|  | No response | 3 | 2.0% |  |

*To what extent do you feel a personal responsibility to try to reduce climate change?*

|  |  | N | % | Valid % |
| --- | --- | --- | --- | --- |
|  | A great deal of responsibility | 60 | 40.0% | 40.5% |
|  | Considerable responsibility | 53 | 35.3% | 35.8% |
|  | Some responsibility | 32 | 21.3% | 21.6% |
|  | Not at all | 3 | 2.0% | 2.0% |
|  | No response | 2 | 1.3% |  |

*Would you be willing to change your own behaviour through the following measures to avoid climate change? (0 - Disagree and 10 - Strongly agree)*

|  |  | N | Min. | Max. | *M* | *SD* |
| --- | --- | --- | --- | --- | --- | --- |
|  | I walk more (less than 3km/2mi) rather than take a car | 149 | 0 | 10 | 7.30 | 2.68 |
|  | I recycle as much waste as possible | 148 | 0 | 10 | 7.58 | 2.59 |
|  | I avoid unnecessary packaging and using plastic bags | 149 | 0 | 10 | 7.58 | 2.61 |
|  | I reduce water use | 150 | 0 | 10 | 7.68 | 2.66 |
|  | I switch off lights when possible | 150 | 1 | 10 | 8.57 | 2.05 |
|  | I buy local products | 149 | 0 | 10 | 7.64 | 2.47 |
|  | I cycle or use public transportation more rather than take a car | 146 | 0 | 10 | 7.18 | 3.00 |
|  | I reduce gas/electricity used in the house | 149 | 0 | 10 | 7.66 | 2.54 |
|  | I take part in a political campaign on an environmental issue | 150 | 0 | 10 | 6.19 | 3.46 |
|  | I change my diet (e.g., vegetarian/vegan/less meat/seasonal food) | 150 | 0 | 10 | 5.77 | 3.47 |
|  | I reduce my consumption (food and other goods) | 148 | 0 | 10 | 6.86 | 2.76 |

*As a religious community, we should do more to address the issues of climate change.*

|  |  | N | % | Valid % |
| --- | --- | --- | --- | --- |
|  | Strongly agree | 73 | 48.7% | 49.0% |
|  | Agree | 61 | 40.7% | 40.9% |
|  | Neither agree nor disagree | 11 | 7.3% | 7.4% |
|  | Strongly disagree | 1 | 0.7% | 0.7% |
|  | I have no opinion | 3 | 2.0% | 2.0% |
|  | No response | 1 | 0.7% |  |

*How interested would you say you are in politics?*

|  | N | % | Valid % |
| --- | --- | --- | --- |
| Very interested | 42 | 28.0% | 28.4% |
| Quite interested | 71 | 47.3% | 48.0% |
| Hardly interested | 22 | 14.7% | 14.9% |
| Not at all interested | 13 | 8.7% | 8.8% |
| No response | 2 | 1.3% |  |

*Do you think religion has a role to play in politics related to climate change?*

|  |  | N | % | Valid % |
| --- | --- | --- | --- | --- |
|  | Yes, very important | 61 | 40.7% | 41.2% |
|  | Yes, important | 55 | 36.7% | 37.2% |
|  | From time to time | 15 | 10.0% | 10.1% |
|  | Rather not | 11 | 7.3% | 7.4% |
|  | Definitely not | 6 | 4.0% | 4.1% |
|  | No response | 2 | 1.3% |  |

*How often have you dealt in the last months in your preaching, religious work or interaction with your congregation/religious followers with the issues of... (1-Never, 2-Hardly ever, 3-Sometimes, 4-Often, 5-Very often)*

|  | Never | | | | Hardly ever | | | | Sometimes | | | | Often | | | | Very often | | | | No response | | |
| --- | --- | --- | --- | --- | --- | --- | --- | --- | --- | --- | --- | --- | --- | --- | --- | --- | --- | --- | --- | --- | --- | --- | --- |
|  | N | % | Valid % | N | | % | Valid % |  | |  | Valid % | N | | % | Valid % | N | | % | Valid % | N | | % | |
| Decrease of biodiversity and that many species (both animals and plants) go extinct | 53 | 35.3 | 35.8 | 35 | | 23.3 | 23.6 | 43 | | 28.7 | 29.1 | 15 | | 10.0 | 10.1 | 2 | | 1.3 | 1.4 | 2 | | | 1.3 |
| Rise of sea levels | 61 | 40.7 | 42.1 | 37 | | 24.7 | 25.5 | 35 | | 23.3 | 24.1 | 8 | | 5.3 | 5.5 | 4 | | 2.7 | 2.8 | 5 | | | 3.3 |
| Weather anomalies (severe droughts, floods, more frequent hurricanes) | 21 | 14.0 | 14.1 | 30 | | 20.0 | 20.1 | 54 | | 36.0 | 36.2 | 31 | | 20.7 | 20.8 | 13 | | 8.7 | 8.7 | 1 | | | 0.7 |
| Issues of poverty and unemployment | 19 | 12.7 | 12.9 | 10 | | 6.7 | 6.8 | 41 | | 27.3 | 27.9 | 47 | | 31.3 | 32.0 | 30 | | 20.0 | 20.4 | 3 | | | 2.0 |
| Large migrations of people following changing climate | 44 | 29.3 | 30.1 | 36 | | 24.0 | 24.7 | 45 | | 30.0 | 30.8 | 16 | | 10.7 | 11.0 | 5 | | 3.3 | 3.4 | 4 | | | 2.7 |
| Too rapid population growth | 66 | 44.0 | 45.5 | 35 | | 23.3 | 24.1 | 23 | | 15.3 | 15.9 | 15 | | 10.0 | 10.3 | 6 | | 4.0 | 4.1 | 5 | | | 3.3 |
| Insufficient food and famines | 23 | 15.3 | 15.6 | 27 | | 18.0 | 18.4 | 37 | | 24.7 | 25.2 | 38 | | 25.3 | 25.9 | 22 | | 14.7 | 15.0 | 3 | | | 2.0 |
| Air and water pollution | 27 | 18.0 | 18.2 | 29 | | 19.3 | 19.6 | 43 | | 28.7 | 29.1 | 30 | | 20.0 | 20.3 | 19 | | 12.7 | 12.8 | 2 | | | 1.3 |
| Excessive consumption | 33 | 22.0 | 22.3 | 15 | | 10.0 | 10.1 | 43 | | 28.7 | 29.1 | 33 | | 22.0 | 22.3 | 24 | | 16.0 | 16.2 | 2 | | | 1.3 |
| Wasting of resources | 32 | 21.3 | 21.8 | 22 | | 14.7 | 15.0 | 41 | | 27.3 | 27.9 | 28 | | 18.7 | 19.0 | 24 | | 16.0 | 16.3 | 3 | | | 2.0 |
| Rise of temperatures | 39 | 26.0 | 26.7 | 24 | | 16.0 | 16.4 | 39 | | 26.0 | 26.7 | 25 | | 16.7 | 17.1 | 19 | | 12.7 | 13.0 | 4 | | | 2.7 |
| Climate change | 33 | 22.0 | 22.3 | 32 | | 21.3 | 21.6 | 41 | | 27.3 | 27.7 | 23 | | 15.3 | 15.5 | 19 | | 12.7 | 12.8 | 2 | | | 1.3 |
