# Supplementary material for: Muslim religious leaders’ views of climate change: An international survey
Source: PLoS One. 2026 Aug 25;21(8):e0353201. doi: 10.1371/journal.pone.0353201 (PMC13505925; doi:10.1371/journal.pone.0353201)
Supplement: Supplementary file 3 [file pone.0353201.s002.docx]

**S2 file. Details and results of the factor analysis of the societal importance of different societal challenges.**

| Communalities | |  |  |
| --- | --- | --- | --- |
|  | Initial | | Extraction |
| climate change | 1.000 | | .735 |
| air and water pollution | 1.000 | | .733 |
| decrease of biodiversity | 1.000 | | .696 |
| excessive consumption | 1.000 | | .689 |
| violent conflicts/crime | 1.000 | | .704 |
| migration | 1.000 | | .631 |
| poverty/unemployment | 1.000 | | .756 |
| corruption | 1.000 | | .691 |
| high taxes | 1.000 | | .713 |
| low education | 1.000 | | .751 |
| lack of universal healthcare/social security | 1.000 | | .694 |
| insufficient food/famine | 1.000 | | .680 |
| **rapid population growth** | **1.000** | | **.394** |
| high disease burden | 1.000 | | .763 |
| drugs/alcohol | 1.000 | | .737 |
| teenage pregnancies | 1.000 | | .807 |
| *Note.* Extraction method: principal component analysis. | |  |  |

Based on the low communality with the other items, we re-ran the analysis excluding the “rapid population growth” item (in bold).

| **Communalities** | | |
| --- | --- | --- |
|  | Initial | Extraction |
| climate change | 1.00 | 0.72 |
| air and water pollution | 1.00 | 0.76 |
| decrease of biodiversity | 1.00 | 0.74 |
| excessive consumption | 1.00 | 0.68 |
| violent conflicts/crime | 1.00 | 0.70 |
| migration | 1.00 | 0.65 |
| poverty/unemployment | 1.00 | 0.76 |
| corruption | 1.00 | 0.69 |
| high taxes | 1.00 | 0.70 |
| low education | 1.00 | 0.74 |
| lack of universal healthcare/social security | 1.00 | 0.69 |
| insufficient food/famine | 1.00 | 0.69 |
| high disease burden | 1.00 | 0.76 |
| drugs/alcohol | 1.00 | 0.76 |
| teenage pregnancies | 1.00 | 0.81 |
| *Note.* Extraction method: principal component analysis. | | |

| **Total variance explained** | | | | | | | | | |
| --- | --- | --- | --- | --- | --- | --- | --- | --- | --- |
| Component | Initial Eigenvalues | | | Extraction sums of squared loadings | | | Rotation sums of squared loadings | | |
|  | Total | % of variance | Cum. % | Total | % of variance | Cum. % | Total | % of Variance | Cum. % |
| 1 | 7.88 | 52.53 | 52.53 | 7.88 | 52.53 | 52.53 | 4.25 | 28.34 | 28.34 |
| 2 | 1.77 | 11.77 | 64.30 | 1.77 | 11.77 | 64.30 | 3.60 | 23.98 | 52.32 |
| 3 | 1.21 | 8.06 | 72.36 | 1.21 | 8.06 | 72.36 | 3.01 | 20.04 | 72.36 |
| 4 | 0.66 | 4.37 | 76.73 |  |  |  |  |  |  |
| 5 | 0.53 | 3.55 | 80.29 |  |  |  |  |  |  |
| 6 | 0.46 | 3.09 | 83.37 |  |  |  |  |  |  |
| 7 | 0.44 | 2.93 | 86.30 |  |  |  |  |  |  |
| 8 | 0.39 | 2.63 | 88.92 |  |  |  |  |  |  |
| 9 | 0.32 | 2.16 | 91.09 |  |  |  |  |  |  |
| 10 | 0.32 | 2.11 | 93.19 |  |  |  |  |  |  |
| 11 | 0.27 | 1.78 | 94.97 |  |  |  |  |  |  |
| 12 | 0.22 | 1.47 | 96.44 |  |  |  |  |  |  |
| 13 | 0.20 | 1.31 | 97.74 |  |  |  |  |  |  |
| 14 | 0.19 | 1.27 | 99.01 |  |  |  |  |  |  |
| 15 | 0.15 | 0.99 | 100.00 |  |  |  |  |  |  |

*Note.* Extraction method: principal component analysis. Rotation method: varimax with Kaiser normalization. Rotation converged in 5 iterations.

| **Component matrix** | | | |  |  |  |
| --- | --- | --- | --- | --- | --- | --- |
|  | Component (Unrotated) | | | Component (Rotated) | | |
|  | 1 | 2 | 3 | 1 | 2 | 3 |
| poverty/unemployment | 0.81 |  |  | 0.81 |  |  |
| insufficient food/famine | 0.79 |  |  | 0.78 |  |  |
| high disease burden | 0.78 |  | -0.38 | 0.76 |  |  |
| lack of universal healthcare/social security | 0.77 |  |  | 0.75 |  |  |
| violent conflicts/crime | 0.77 |  |  | 0.74 |  |  |
| corruption | 0.76 |  |  | 0.73 |  |  |
| high taxes | 0.76 |  |  |  | 0.84 |  |
| drugs/alcohol | 0.74 |  | -0.43 |  | 0.81 |  |
| low education | 0.74 |  |  |  | 0.74 | 0.38 |
| teenage pregnancies | 0.73 |  | -0.42 | 0.38 | 0.73 |  |
| migration | 0.73 |  |  | 0.47 | 0.65 |  |
| decrease of biodiversity | 0.69 | 0.45 |  |  |  | 0.84 |
| air and water pollution | 0.58 | 0.65 |  |  |  | 0.81 |
| excessive consumption | 0.54 | 0.61 |  |  |  | 0.80 |
| climate change | 0.60 | 0.60 |  |  | 0.46 | 0.71 |

*Note.* Extraction method: principal component analysis. Rotation method: varimax with Kaiser normalization. Rotation converged in 5 iterations.

| **Component transformation matrix** | | | |
| --- | --- | --- | --- |
| Component | 1 | 2 | 3 |
| 1 | 0.67 | 0.59 | 0.45 |
| 2 | -0.36 | -0.27 | 0.89 |
| 3 | 0.65 | -0.76 | 0.03 |

*Note.* Extraction method: principal component analysis. Rotation method: varimax with Kaiser normalization.
