# Supplementary material for: Muslim religious leaders’ views of climate change: An international survey
Source: PLoS One. 2026 Aug 25;21(8):e0353201. doi: 10.1371/journal.pone.0353201 (PMC13505925; doi:10.1371/journal.pone.0353201)
Supplement: Supplementary file 5 [file pone.0353201.s004.docx]

**S4 file. Descriptive statistics for selected study variables by survey mode.**

Values are presented for descriptive purposes only. Because survey mode was confounded with geographic region and respondents were not randomly assigned to survey modes, differences between online and paper respondents should not be interpreted as evidence of survey-mode effects.

|  |  | Paper/pencil | Online | Total |
| --- | --- | --- | --- | --- |
| N |  | 71 | 79 | 150 |
| *Do you think the world's climate is changing?* (%) | | | | |
|  | Definitely | 66.2 | 80.8 | 73.8 |
|  | Probably | 32.4 | 17.9 | 24.8 |
|  | Probably not | 1.4 | 0.0 | 0.7 |
|  | No opinion | 0.0 | 1.3 | 0.7 |
| *Do you think climate change is caused by natural processes, human activity, or both?* (%) | | | | |
|  | Entirely natural processes | 0.0 | 1.4 | 0.7 |
|  | Mainly natural processes | 1.4 | 9.9 | 5.6 |
|  | About equally natural processes and human activity | 39.4 | 38.0 | 38.7 |
|  | Mainly human activity | 39.4 | 49.3 | 44.4 |
|  | Entirely human activity | 12.7 | 1.4 | 7.0 |
|  | No opinion | 7.0 | 0.0 | 3.5 |
| Importance of the environment as a societal challenge, M (SD)  0=no importance; 10=very high importance | | 7.29 (2.08) | 7.43 (1.94) | 7.36 (2.00) |
| *Muslim religious leaders should be more active in actions related to climate change.* | | | | |
|  | Strongly agree | 47.9 | 53.8 | 51.0 |
|  | Agree | 32.4 | 33.3 | 32.9 |
|  | Neither agree nor disagree | 14.1 | 6.4 | 10.1 |
|  | Disagree | 2.8 | 1.3 | 2.0 |
|  | Strongly disagree | 1.4 | 1.3 | 1.3 |
|  | No opinion | 1.4 | 3.8 | 2.7 |
| Willing to change own behavior to avoid climate change, M (SD)  0=not at all willing; 10=highly willing | | 7.85 (1.76) | 6.75 (1.98) | 7.27 (1.95) |
| Willingness to promote mitigation strategies, median (IQR)  1=yes, strongly; 5=absolutely not | | | | |
|  | Limiting population growth/family size | 3.00 (3.00) | 4.00 (3.00) | 3.00 (3.00) |
|  | Reducing consumption | 2.00 (1.00) | 2.00 (2.00) | 2.00 (1.00) |
|  | Changing dietary habits, less meat consumption | 2.00 (2.00) | 2.00 (3.00) | 2.00 (2.00) |
|  | Vegetarianism | 3.00 (2.00) | 3.00 (3.00) | 3.00 (3.00) |
|  | Implementation of new technologies for greener production/consumption | 1.00 (1.00) | 2.00 (2.00) | 2.00 (1.00) |
|  | Government spending for technology that reduces emissions/curb effects of climate change | 1.00 (1.00) | 2.00 (2.00) | 2.00 (1.00) |

*Note.* Sample sizes vary slightly across variables because of missing data; all statistics are based on valid cases. Percentages may not sum to 100% because of rounding. IQR = interquartile range*.*
