# Supplementary material for: Muslim religious leaders’ views of climate change: An international survey
Source: PLoS One. 2026 Aug 25;21(8):e0353201. doi: 10.1371/journal.pone.0353201 (PMC13505925; doi:10.1371/journal.pone.0353201)
Supplement: Supplementary file 7 [file pone.0353201.s006.docx]

Muslim religious leaders’ views of climate change: An international survey.

**Additional Comment-**

1. Around 150 Muslim religious leaders’ Precipitant and give their feedback but totally Unclear (Need more information about them). You need to add and the total number of participants from each country should be shown in a separate table.
2. Any differences between paper and online participants should be analyzed and noted.
3. You need to add a complete copy of the questionnaire and it should be attached.
4. It would be very helpful for readers and the researcher the language should be more sensitive and neutral in discussions of religion and demographics.

**Comment**

Although the study addresses an important issue, it has several fundamental methodological shortcomings. There is a risk of bias due to the use of possessive and snowball sampling, the number of people invited, and how many responded. Statistical analysis appears to be performed to maintain general standards, but important information is missing. The full procedure for factor analysis is not described.

No confidence intervals were added for Cronbach's alpha, and no potential differences were analyzed between paper-based and online participants, which were very important.

The authors have not disclosed the complete data in this study. The authors mention the availability of OSF data and SPSS, but the link provided cannot be confirmed as to whether the data set is complete or not. The Raw data, court book, and complete analysis scripts need to be released for re-evaluation. The data currently provided is not up to standard.

The research is fairly understandable, but there is Scope for improvement. The language used in discussions of religion and demographics should be both neutral and sensitive. The caption of each figure is not clear; the axis name, scale, and sample size should be mentioned. Find this study incomplete because there are some missing questions. However, with some corrections, this study can be made readable.
