# Supplementary material for: Muslim religious leaders’ views of climate change: An international survey
Source: PLoS One. 2026 Aug 25;21(8):e0353201. doi: 10.1371/journal.pone.0353201 (PMC13505925; doi:10.1371/journal.pone.0353201)
Supplement: Supplementary file 8 [file pone.0353201.s005.docx]

Response to Editor' Comments

Reviewer (Remarks to the Author):

It is very interesting that the author combines religious culture with climate change. However, the analysis remains superficial and lacks an in-depth exploration of the underlying reasons.

1. Please add keywords to the manuscript.

2. There is limited literature review on the impact of religious leaders on climate change, with the author citing only one reference.

3. The author only superficially describes the influence of religious leaders on climate change without providing an in-depth analysis of the underlying reasons. It is recommended to discuss these reasons in the discussion section.

4. It is advisable to improve the clarity of the figure.
