# Supplementary material for: Muslim religious leaders’ views of climate change: An international survey
Source: PLoS One. 2026 Aug 25;21(8):e0353201. doi: 10.1371/journal.pone.0353201 (PMC13505925; doi:10.1371/journal.pone.0353201)
Supplement: Supplementary file 9 [file pone.0353201.s008.docx]

**Response to Editor and Reviewers [PONE-D-25-52492]**

Following consultation with the PLOS ONE editorial staff (Case No. 09388620), we were advised that a response to Reviewer 1’s comments was not required. We have therefore focused our efforts on addressing the remaining editor and reviewer comments.

**_______________________________________________________________________________________**

**Editor**

*The most critical issues that must be addressed are:
1. There are significant concerns regarding the sampling strategy, data collection procedures, and statistical reporting. A more detailed justification of the methods and enhanced transparency are required.*

We have addressed the reviewers’ concerns in the point-by-point rebuttal below. Briefly, we have elaborated on the limitations of our sampling strategy and described some additional limitations; provided information about the translation procedure; and provided more information about the statistical approach. The questionnaire, raw data, and SPSS data analysis scripts are available on the Open Science Foundation (OSF) repository. Below, line numbers refer to the version of the revised manuscript with Track Changes.

*2. The introduction needs to more clearly articulate the specific research gap this study fills, distinguishing it from existing literature and explicitly stating its novelty.*

We have re-written the Introduction to make the aim and novelty of the study more explicit. We now write on lines 78 -83: *In the current study, we provide quantitative evidence on Muslim religious leaders’ beliefs about the causes and consequences of climate change, their perceptions of the role of Muslim leaders in addressing climate change, and their willingness to personally engage in and promote climate mitigation behaviors. Few studies have examined religious leaders’ climate views, and most existing research has been either qualitative or focused primarily on Christian leaders. By analyzing survey data from Muslim religious leaders, this study addresses an important gap in the literature.*

*3. The results section is currently largely descriptive. A deeper interpretation of the findings, linking them to theoretical frameworks and contextual factors, is necessary.*

We have aimed to adhere to PLOS ONE guidelines. According these guidelines, authors may choose to present their results and discussion either separately or in a combined “Results and Discussion” section. We have chosen to present them separately, with the Results section focusing on objective presentation of the findings and interpretation provided in the Discussion section. To ensure this distinction is clear, we have reviewed the manuscript and strengthened the interpretive components in the Discussion where appropriate.

*4. The manuscript requires thorough copyediting to improve grammar, sentence flow, and overall clarity. Figures and captions must be made self-explanatory and consistent.*

We have thoroughly proofread the manuscript and revised several passages.

*Specific Required Revisions:*

*Please address the following points, which synthesize and prioritize the concerns raised by all reviewers:*

*1. Methodological Clarifications and Enhancements (Primarily from Reviewers 1, 2, & 3):
Sampling Bias: Acknowledge the limitations of the convenience and snowball sampling method more explicitly. Discuss potential biases (e.g., over-representation of climate-engaged leaders) and how they might affect the generalizability of the findings. While increasing the sample size (as suggested by Reviewer 2) is not feasible post-hoc, a more nuanced discussion of this limitation is essential.*

We have now elaborated on the limitations of our sampling method, sample size, and potential biases in the Strengths, limitations, and future research section (lines 371-395).

*Participant Demographics: Provide a detailed table (as suggested by Reviewer 3) summarizing the number of participants from each country, their roles (e.g., imam, teacher), school of jurisprudence, and mode of survey completion (online vs. paper).*

We have now moved the descriptive statistics from the main text into a dedicated table (Table 1), which summarizes the number of participants by country, their roles (e.g., imam, teacher), school of jurisprudence, and mode of survey completion (online vs. paper). The table also includes additional relevant demographic characteristics for completeness.

*Survey Instrument and Translation: Clarify the procedures used for translating and validating the questionnaire across multiple languages (e.g., back-translation, expert review) to ensure conceptual equivalence (Reviewer 5).*

We have now clarified the translation procedure in the Methods/Procedure section (lines 118-122). While we did not conduct formal statistical tests of measurement equivalence across language versions, the items consisted primarily of direct and concrete questions rather than culturally-nuanced or complex constructs. Nevertheless, we recognize that the absence of formal equivalence testing is a limitation and have now acknowledged this explicitly in the manuscript (lines 388-390).

*Factor Analysis: Provide a complete description of the factor analysis in the main text or the supplementary materials. This must include the extraction method (e.g., Principal Component Analysis), rotation method, Kaiser-Meyer-Olkin (KMO) measure, Bartlett's test of sphericity results, and a full table of factor loadings (Reviewer 5).*

In the main text, we have clarified that we conducted a principal components analysis (PCA) with orthogonal varimax rotation and Kaiser normalization (lines 146-147). Further details and the complete results of the analysis - including the extraction method, rotation method, KMO measure, Bartlett’s test of sphericity, and the full table of component loadings - are provided in the S2 file. To ensure clarity, we have made the reference to the S2 file more explicit (line 154-155).

*Statistical Analysis: Justify the use of parametric tests (t-tests) on Likert-type data, citing relevant methodological literature on treating such scales as interval data (Reviewer 5).*

The manuscript includes a citation to methodological literature supporting the use of parametric tests with 11-point Likert-type scales; we have revised the text to make this justification more explicit (lines 179-180).

*Report confidence intervals for Cronbach's alpha values (Reviewer 3).*

We have added 95% confidence interval for the Cronbach's alpha values on (lines 156-158 and 166). We have likewise amended the SPSS script on the OSF repository.

*Conduct and report a comparative analysis between paper-based and online respondents to check for mode effects (Reviewer 3).*

We employed a mixed-mode design (paper-based and online questionnaires) to maximize coverage and accessibility across regions and linguistic groups. Mixed-mode approaches can enhance inclusivity and improve reach, particularly when sampling geographically dispersed or hard-to-access populations.

However, in our sample, mode of data collection is significantly associated with region of residence (χ²(4, N = 149) = 38.37, p < .001; see cross-tabulation below). This creates a structural confound between survey mode and region. Consequently, any observed differences between paper-based and online respondents could reflect regional variation rather than mode effects per se, rendering direct comparisons ambiguous.

Moreover, subsample sizes within regions are insufficient for adequately powered within-region mode comparisons. For example, detecting a medium effect (Cohen’s d = 0.5) with α = .10 and 80% power would require approximately 51 participants per mode within each region, which is not met in the present data. Within several regions, mode is additionally confounded with country of residence.

For these reasons, we do not consider a comparative analysis between modes to yield interpretable results. Nevertheless, we agree with the reviewers that survey mode may have influenced responses. We have now explicitly noted this as a limitation in the Strengths, limitations, and future research section (lines 390-395).

| **Paper/pencil or online questionnaire * world region of residence Crosstabulation** | | | | | | | |
| --- | --- | --- | --- | --- | --- | --- | --- |
| Count | | | | | | | |
|  | | world region of residence | | | | | Total |
|  |  | Western Europe and USA | post-Soviet | MENA | sub-Saharan Africa | Asia |  |
| paper/pencil or online questionnaire | paper/pencil | 15 | 0 | 13 | 22 | 20 | 70 |
|  | online | 17 | 17 | 31 | 6 | 8 | 79 |
| Total | | 32 | 17 | 44 | 28 | 28 | 149 |

*2. Introduction and Theoretical Framing (Primarily from Reviewers 1 & 5):
Research Gap: Sharply define the research gap in the introduction. Explicitly state how this study moves beyond existing theoretical work on Islamic environmental ethics and limited surveys of Christian leaders.*

We agree with the reviewers that the research gap and the study’s contribution needed to be more sharply articulated. We have therefore revised the Introduction to make the aim and novelty of the study more explicit. We now write on lines 78 -83: *In the current study, we provide quantitative evidence on Muslim religious leaders’ beliefs about the causes and consequences of climate change, their perceptions of the role of Muslim leaders in addressing climate change, and their willingness to personally engage in and promote climate mitigation behaviors. Few studies have examined religious leaders’ climate views, and most existing research has been either qualitative or focused primarily on Christian leaders. By analyzing survey data from Muslim religious leaders, this study addresses an important gap in the literature.*

*Theoretical Framework: Strengthen the theoretical underpinnings by integrating relevant frameworks such as elite cue theory, moral foundations theory, or value-belief-norm theory to better frame why religious leaders' views matter (Reviewer 5).*

We agree with the reviewer that the theoretical underpinnings of the study could be strengthened. In response, we have revised the first paragraph of the Introduction to explicitly reference theoretical and empirical work on the role of elite cues, moral values, and social identities in shaping climate beliefs and behaviors, thereby reinforcing why religious leaders’ views are important. We have also added seven new references to support this framing.

*Literature Review: Integrate the suggested literature (e.g., Koehrsen, 2021; Mazaheri, 2024) to better contextualize the study within the emerging field of Islam and climate change.*

We appreciate the literature suggestions. In response, we have added a reference to Mazaheri (2024) in the Discussion section (reference #30, line 288). Koehrsen (2021) was already cited in the manuscript (reference #34; lines 320 and 366).

*3. Results and Discussion (Primarily from Reviewers 1, 3, & 5):
Beyond Description: The results section should not only report findings but also provide initial interpretation and highlight key patterns. Avoid simply restating what is evident in the figures and tables.*

We aimed to adhere to PLOS ONE guidelines, which state that authors may choose to present their results and discussion either separately or in a combined “Results and Discussion” section. We have chosen to present them separately, with the Results section focusing on objective presentation of the findings and interpretation provided in the Discussion section. To ensure this distinction is clear, we have reviewed the manuscript and strengthened the interpretive components in the Discussion where appropriate.

*Nuanced Discussion: The discussion should more deeply explore the nuances of the findings. For example:*

*i. When comparing results with Christian clergy studies, discuss the distinct political, cultural, and theological contexts that may explain similarities or differences (Reviewer 5).*

We appreciate the suggestion to discuss what might explain why both the Muslim leaders in our study, and the (mostly) Christian leaders in the study from Syropoulos and Sparkman acknowledge human-caused climate change but do not meaningfully address it in their work as religious leaders. We now write (lines 306-315): *The observation that both Muslim leaders in the current study and primarily Christian leaders in the study from Syropoulos and Sparkman (14) acknowledge anthropogenic climate change yet rarely address it in their religious work suggests convergence in behavior, but not necessarily the reasons for it. Some reasons may cut across faith groups, such as deprioritizing climate change in the face of more immediate concerns (providing moral or spiritual guidance; supporting community members during crises), or having limited guidance on how to meaningfully integrate environmental issues into their religious work. Other reasons may be specific to religious leaders of particular faiths or in specific social contexts. Future research should examine why religious leaders do not address climate change in their religious work, recognizing that the reasons may potentially vary across theological traditions, institutional structures, political contexts, and geographies.*

*ii. Elaborate on the potential reasons behind the reluctance to promote dietary changes and fertility limitation, moving beyond speculation to a more critical analysis grounded in the literature on Islamic ethics and demographic debates.*

We appreciate the reviewer’s suggestion to elaborate on potential reasons behind respondents’ reluctance to promote dietary change and fertility limitation. Our study did not collect data on these underlying motivations, so any discussion would necessarily be speculative and rely on existing literature. Additionally, we do not have specialized training in Islamic jurisprudence or ethical theory; providing a detailed theological or jurisprudential analysis would fall outside the appropriate scope of this manuscript and could risk overstating our expertise.

In response to the reviewer’s suggestions, we now highlight the need for future research that integrates empirical investigation with a critical engagement with the literature on Islamic ethics to better understand why Muslim religious leaders may be reluctant to promote dietary change and fertility limitation as strategies for mitigating climate change (lines 402-405).

*iii. Policy Implications: The conclusion should be concise and include clear, actionable policy implications derived from the findings, as suggested by Reviewer 1.*

We have not addressed Reviewer 1’s comments in this revision following consultation with the PLOS ONE editorial staff (Case No. 09388620).

We note that our findings are based on a convenience sample of N=150 Muslim religious leaders from diverse cultural and social contexts. Given the modest sample size and substantial heterogeneity across contexts, deriving specific, actionable policy recommendations would overextend the data. We do, however, suggest that interventions aimed at enhancing Muslim religious leaders’ self-efficacy to engage in political campaigns may help unlock their potential to motivate climate-friendly policies and community-wide action (lines 336-338). In the Strengths, limitations, and future research section, we now explicitly note that the non-representative and heterogeneous nature of our sample precludes generalizable policy recommendations (lines 382-384).

*4. Data and Code Availability (Primarily from Reviewer 3):
Full Data Disclosure: Ensure that the data provided via the OSF link constitutes the complete, de-identified dataset used for all analyses in the manuscript. This should include the raw data, the codebook variable definitions, and the complete SPSS (or other software) syntax scripts used for data cleaning and analysis. The current statement must be verified and confirmed.*

We appreciate the importance of transparency. The OSF repository includes the raw data, the complete SPSS syntax scripts, a codebook with variable definitions, and the questionnaire. The wording of all questionnaire items analyzed in the current study remains available in the S1 file. We confirm that the OSF repository contains the complete, de-identified dataset used for all analyses reported in the manuscript.

*5. Language and Presentation (All Reviewers):
Professional Copyediting: The manuscript must undergo thorough professional English language editing to correct grammatical errors, improve sentence construction, eliminate redundancy, and enhance overall readability.*

We have thoroughly proofread the manuscript and revised several passages to improve readability. We note that concerns about readability were raised only by Reviewer 1. All reviewers, including Reviewer 1, indicated that the manuscript is intelligible and written in standard English.

*Figures and Captions: Ensure all figures are high-resolution and that all captions are uniform, self-explanatory, and explicitly mention the sample size (N=150). Each axis must be clearly labeled, and the caption should fully describe what the figure depicts without requiring the reader to refer back to the main text (Reviewers 1 & 3).*

We have now ensured that the figures meet the requirements of the journal.

**Comments to the Author**

1. Is the manuscript technically sound, and do the data support the conclusions?

Reviewer #1: Yes

Reviewer #2: Yes

Reviewer #3: Partly

Reviewer #4: Yes

Reviewer #5: Yes

2. Has the statistical analysis been performed appropriately and rigorously?

Reviewer #1: Yes

Reviewer #2: Yes

Reviewer #3: No

Reviewer #4: Yes

Reviewer #5: Yes

*3. Have the authors made all data underlying the findings in their manuscript fully available?*

*Reviewer #1: Yes*

*Reviewer #2: Yes*

*Reviewer #3: No*

*Reviewer #4: Yes*

*Reviewer #5: Yes*

**Response:** All data underlying the findings are provided in the Supplementary Files and the OSF repository submitted with the manuscript, including the raw dataset, SPSS syntax, codebook, and questionnaire.

4. Is the manuscript presented in an intelligible fashion and written in standard English?

Reviewer #1: Yes

Reviewer #2: Yes

Reviewer #3: Yes

Reviewer #4: Yes

Reviewer #5: Yes

**Reviewer #1**

*Various articles for improvement

1. Koehrsen, J. (2021). Muslims and climate change: How Islam, Muslim organizations, and religious leaders influence climate change perceptions and mitigation activities. Wiley Interdisciplinary Reviews: Climate Change, 12(3), e702.

2. Skirbekk, V., & Pędziwiatr, K. (2018). Sustainability and climate change in major religions with a focus on Islam. Humanitarian Academy for World Development Research Paper.

3. Mazaheri, N. (2024). Faith in science: religion and climate change attitudes in the Middle East. Global Environmental Politics, 24(1), 52-75.

4. Ali, A., Ali, S., Baird, I. G., & Yaseen, M. (2025). Exploring the potential role of Muslim spiritual leaders in coping with climate change through tree planting in Pakistan. Local Environment, 30(4), 520-533.

5. Agusalim, L., & Karim, M. (2024). Religiosity and climate change: An eco-religious approach. Environmental & Socio-Economic Studies, 12(1), 35-50.*

**Response:** We have not responded to Reviewer 1’s comments in this revision following consultation with the PLOS ONE editorial staff (Case No. 09388620). We have, however, reviewed the suggested references. References 1 and 2 were already included in the manuscript, and we have now added references 3 and 5. After careful consideration, we determined that reference 4 falls outside the scope of the current study and have therefore not included it.

__________________________________________________________________________________

**Reviewer #2**

*It would have been great. If the author could have further increased the weightage of the paper by increasing the sample size from 150 to 1500 or as maximum as possible. However the manuscript is accepted in the current form.*

**Response:** We appreciate the reviewer’s positive assessment. We see our study as a first impression of Muslim leaders’ climate views, and hope it encourages future research with larger and more representative samples.

**Reviewer #3**

We thank Reviewer 3 for their thoughtful comments. Below, line numbers refer to the version of the revised manuscript with Track Changes.

*Although the study addresses an important issue, it has several fundamental methodological shortcomings. There is a risk of bias due to the use of possessive and snowball sampling, the number of people invited, and how many responded. Statistical analysis appears to be performed to maintain general standards, but important information is missing. The full procedure for factor analysis is not described.*

We appreciate that the reviewer recognizes our study addresses an important and timely topic. We view this work as a first impression of Muslim religious leaders’ climate-related views. We acknowledge the relatively small sample size and the limitations of our sampling approach, and we have now elaborated on these (and other) limitations in the Strengths, limitations, and future research section. In the main text, we have now clarified that we ran a principal components analysis with orthogonal varimax rotation and Kaiser normalization (lines 146-147). Further details and the complete results of the principal components analysis, including extraction and rotation methods, KMO measure, Bartlett’s test of sphericity, and component loadings, remain available in the S2 file.

*No confidence intervals were added for Cronbach's alpha, and no potential differences were analyzed between paper-based and online participants, which were very important.*

We thank the reviewer for their careful reading. We have added confidence intervals for Cronbach’s alpha values (lines 156-158 and 166). We have likewise amended the SPSS script on the OSF repository. Regarding differences between paper-based and online participants, please see our detailed response below.

*The authors have not disclosed the complete data in this study. The authors mention the availability of OSF data and SPSS, but the link provided cannot be confirmed as to whether the data set is complete or not. The Raw data, court book, and complete analysis scripts need to be released for re-evaluation. The data currently provided is not up to standard*.

We appreciate the reviewer’s focus on transparency. All materials underlying the analyses are available in the Supplementary Files and the OSF repository, including the raw data, the complete SPSS syntax scripts, and the wording of all study items. To further enhance transparency, we have now added a codebook and the questionnaire to the OSF repository. We confirm that the dataset is complete and constitutes all data used in the analyses reported in the manuscript. We are open to any additional suggestions for further improving transparency.

*The research is fairly understandable, but there is Scope for improvement. The language used in discussions of religion and demographics should be both neutral and sensitive.*

We appreciate the reviewer’s attention to the sensitivity and neutrality of language. While no specific examples of where we should adapt the language were provided, we have carefully reviewed the manuscript and ensured that all discussions of religion and demographics focus on empirical findings - both our own and those from previous studies -without making normative or evaluative statements.

*The caption of each figure is not clear; the axis name, scale, and sample size should be mentioned. Find this study incomplete because there are some missing questions. However, with some corrections, this study can be made readable.*

We have ensured that the axes and scale of all figures are clearly labelled. The sample size (N=150 Muslim religious leaders) is stated in all of the figure captions.

*Additional comments-*

*1. Around 150 Muslim religious leaders’ Precipitant and give their feedback, but it is totally Unclear (Need more information about them). You need to add the total number of participants from each country to a separate table.*

We have now moved the descriptive characteristics of the sample from the main text to a dedicated table (Table 1). To provide a clear overview of the sample, Table 1 includes data on twelve characteristics, including the total number of participants from each country, their roles, school of jurisprudence, and mode of survey completion.

*2. Any differences between paper and online participants should be analyzed and noted.*

We thank the reviewer for this comment. We employed a mixed-mode design to maximize coverage and accessibility across regions and linguistic groups. Mixed-mode approaches can enhance inclusivity, particularly when sampling geographically dispersed or hard-to-access populations.

In our sample, mode of data collection is significantly associated with region of residence (χ²(4, N = 149) = 38.37, p < .001; see cross-tabulation above), creating a structural confound between survey mode and region. Consequently, observed differences between paper-based and online respondents could reflect regional variation rather than mode effects, making direct comparisons ambiguous. Subsample sizes within regions are also insufficient for adequately powered within-region comparisons; for example, detecting a medium effect (Cohen’s d = 0.5) with α = .10 and 80% power would require approximately 51 participants per mode within each region, which is not met in the present data. Within several regions, mode is additionally confounded with country of residence. We therefore feel that comparative analyses of the online versus offline respondents are not interpretable in the current data set.

In response to the reviewer’s comment, we now acknowledge the potential influence of survey mode on our results, while clarifying why comparative analyses are not interpretable in the current dataset in the Strengths, Limitations, and Future Research section (lines 390–395): *We collected data using both offline and online questionnaires. Online and paper questionnaires share several key characteristics (e.g., they are both self-administered and visually mediated), and empirical research suggests they are often yield comparable results (48). However, survey responses do sometimes vary depending on whether a questionnaire is completed online or offline (49). Unfortunately, our data does not allow us to disentangle mode effects from geographical variation, and it is hence unclear whether the mode of data collection affected the responses.*

We also call for future research that investigates potential survey mode effects (line 399-400).

*3. You need to add a complete copy of the questionnaire, and it should be attached.*

We appreciate the reviewer’s attention to transparency. All of the questionnaire items analyzed in the study remain in the S1 file. In response to this comment, we have now also added a copy of the complete questionnaire to the OSF repository to enhance accessibility and transparency.

*4.It would be very helpful for readers and the researcher if the language were more sensitive and neutral in discussions of religion and demographics.*

As we stated earlier, we appreciate the reviewer’s attention to the sensitivity and neutrality of language. While no specific examples of where we should adapt the language were provided, we have carefully reviewed the manuscript and ensured that all discussions of religion and demographics focus on empirical findings - both our own and those from previous studies -without making normative or evaluative statements. We would be happy to address any specific examples the reviewer may identify if they feel further revisions are necessary.

__________________________________________________________________________________

**Reviewer #4**

*The manuscript obtained data through a questionnaire survey, described a technically reasonable scientific research, and provided data to support the conclusion. For instance, the author presented through us the results of a global survey of Muslim religious leaders (N=150) from 29 countries, such as Imams, religious teachers, and students of religious colleges. Our research findings indicate that the majority of respondents believe that climate change is man-made and that it has already caused serious harm. Participants also highly agreed that the Muslim religious community should make more efforts to address climate change. Islam provides a set of values and principles that encourage environmentally responsible behavior. Respondents reported that they sometimes involved climate change and other environmental issues in their religious work. They expressed their strong willingness to encourage the government to invest in technologies aimed at curbing emissions and the impacts of climate change. Adopt new technologies to promote more environmentally friendly production and consumption; Reduced consumption. There is a relatively low willingness to promote adjustments in dietary habits and reduce meat consumption. Vegetarianism Or limit population growth and family size. The logic is clear and the conclusion is definite. It is an article worth discussing. At the same time, appropriate and strict statistical analysis was carried out. In addition to summary statistics, data points behind mean, median and variance measures should also be provided. The writing of the manuscript is also relatively easy to understand, and the English writing is also quite standardized.*

**Response:** We appreciate the reviewer’s positive assessment. The complete data are available in the OSF repository.

__________________________________________________________________________________

**Reviewer #5**

*This paper discusses the important issue of climate-change attitudes among Muslim religious leaders globally. The Paper has broader reader interest and may be published after addressing the following issues:*

We appreciate the reviewer’s positive evaluation and constructive feedback. Below, line numbers refer to the revised version of the manuscript with Track Changes enabled.

*1. Page 1 Lines 18–31 (Abstract) The abstract is clear but overly descriptive. The abstract provides a theoretical explanation for the significance of Muslim religious leaders, citing factors such as moral authority and social capital.*

We agree that the Abstract was overly descriptive and have revised it to include some key, specific results. Because some sentences summarize findings across multiple measures, we do not provide statistics for each result.

*2. Page 2 Lines 33–44 The introduction states the importance of religious leaders but could benefit from clearer theoretical framework (e.g., moral foundations, social identity, and elite cue theory).
3. Page 3 Line 41 The introduction states the importance of religious leaders but could benefit from clearer theoretical framework (e.g., moral foundations, social identity, and elite cue theory).*

We note that Points 2 and 3 are identical and thank the reviewer for this suggestion. In the first paragraph of the Introduction, we now refer to theoretical and empirical work on the importance of elite cues, moral values, and social identities as drivers of climate beliefs and behaviors, thereby strengthening our argument that religious leaders’ views matter. We have also added seven new references to support this framing.

*4. Page 3 Line 42 “wary of science…in particular.” This is accurate but needs nuance to avoid suggesting religious people are uniformly anti-science.*

We thank the reviewer for this comment. The phrase “wary of science…in particular” refers specifically to conservative groups, not religious people in general. For enhanced clarity, we have added a parenthetical comment and a reference explicitly acknowledging that religious belief is not inherently at odds with confidence in science (lines 53-54).

*5. Page 3 Line 66 representing why Muslim leaders are uniquely understudied.*

We are unsure about the concern, as Page 3, Line 66 referenced a study specifically on Christian clergy. We would be happy to clarify this sentence if the reviewer has specific suggestions.

*6. Page 4-5 line 101-105 Useful but explain how this relates to your analyses. Did you test urban/rural differences? If not, move this to descriptive table.*

The second paragraph of the “Sample” section provided various descriptive statistics for our participants. In response to this comment, we have moved all of the information in this paragraph to a dedicated table (Table 1) to improve clarity.

*7. Page 6 Lines 110–113 Because the questionnaire was administered in multiple languages, please clarify whether translations were checked (e.g., via back-translation or expert review). This ensures consistency across survey modes.*

We thank the reviewer for this important comment. We have now clarified the translation procedure in the Methods section (lines 118-122): *To increase the potential sample size, we developed an online version of the survey and translated it into additional languages. English served as the master version, and the questionnaire was forward-translated into Arabic, French, Russian, and Turkish with the assistance of bilingual collaborators. No back-translation or formal validation procedures were employed.*

We acknowledge this as a limitation (lines 388-390): *No back-translation or formal validation procedures were conducted for the translated survey instruments, which may have introduced minor inconsistencies across language versions, although the items were relatively straightforward and seem unlikely to vary substantially across cultural contexts.*

*8. Page 5 lines 118–133 Only minimal information is provided. Please add the extraction method (e.g., principal components or maximum likelihood) and KMO measure and Bartlett’s test*

We now state clearly state that we conducted a principal components analysis (PCA) with orthogonal varimax rotation and Kaiser normalization (lines 146-147). Further details and the full results of the principal component analysis - including the KMO measure, Bartlett’s test of sphericity, and component loadings - are provided in the S2 file. This supplementary file contains all of the information requested and allows readers to fully evaluate the principal component analysis conducted in the study.

*9. Page 6 line 148-150 statistical methods are straightforward, but justify why parametric tests were used with 11-point Likert items, but still useful to mention its robustness.*

The manuscript includes a citation to methodological literature supporting the use of parametric tests with 11-point Likert-type scales; we have revised the text to make this justification more explicit (lines 179-180).

*10. Page 9 Lines 263-271 Avoid overstating consistency with Christian studies; Muslim contexts differ culturally and politically. Add nuance: differences in democratic participation, political risks, etc.*

We thank the reviewer for this comment, which refers to the comparison of our findings with a large study of primarily Christian clergy in the United States. Our comparison refers specifically to empirical convergence: both studies find that religious leaders acknowledge anthropogenic climate change but tend not to address it in their religious work. We do not draw broader normative or contextual conclusions beyond this empirical parallel. Both Christian and Muslim contexts are highly diverse, with substantial variation in cultural, political, and social factors within each. For example, electoral participation varies widely in both Muslim and Christian settings: turnout is relatively high in Indonesia (Muslim-majority) and Germany (Christian-majority), and relatively low in Jordan (Muslim-majority) and the United Kingdom (Christian-majority). We therefore remain cautious about assuming systematic differences between Christian and Muslim contexts and focus strictly on findings grounded in empirical evidence.

In response to your comment, we have clarified that our comparison refers specifically to the empirical finding that both Muslim and Christian religious leaders acknowledge anthropogenic climate change but do not consistently address it in their religious work. We also emphasize that, despite this behavioral similarity, the underlying reasons for such inaction may differ across theological, cultural, and political contexts (lines 306-315): *The observation that both Muslim leaders in the current study and primarily Christian leaders in the study from Syropoulos and Sparkman (14) acknowledge anthropogenic climate change yet rarely address it in their religious work suggests convergence in behavior, but not necessarily the reasons for it. Some reasons may cut across faith groups, such as deprioritizing climate change in the face of more immediate concerns (providing moral or spiritual guidance; supporting community members during crises), or having limited guidance on how to meaningfully integrate environmental issues into their religious work. Other reasons may be specific to religious leaders of particular faiths or in specific social contexts. Future research should examine why religious leaders do not address climate change in their religious work, recognizing that the reasons may potentially vary across theological traditions, institutional structures, political contexts, and geographies.*

We thank the Editor and the reviewers for their careful evaluation of our manuscript and for their constructive feedback!
