# Supplementary material for: Muslim religious leaders’ views of climate change: An international survey
Source: PLoS One. 2026 Aug 25;21(8):e0353201. doi: 10.1371/journal.pone.0353201 (PMC13505925; doi:10.1371/journal.pone.0353201)
Supplement: Supplementary file 10 [file pone.0353201.s010.docx]

Dear Dr. Raimi,

Thank you for your careful evaluation of our manuscript and for the opportunity to submit a revised version.

We have revised the manuscript in response to the three points raised in your decision letter. Specifically, we (1) added a bridging sentence in the Introduction to further clarify the study's descriptive aim, (2) revised the discussion of survey mode effects in the limitations section to emphasize the confounding of survey mode and geographic region, and (3) added descriptive statistics for six study constructs by survey mode to the Supporting Information, accompanied by an explicit note regarding the limitations of interpreting these comparisons.

In addition, we reviewed the reference list and corrected several bibliographic details (e.g., DOIs, URLs) to ensure completeness and accuracy. We confirmed that none of the cited references have been retracted.

We have included both a clean version of the manuscript and a tracked-changes version, as well as a detailed point-by-point response describing all revisions.

We appreciate your constructive feedback throughout the review process and hope that the revised manuscript is now suitable for publication in PLOS One.

Sincerely,

Catherine E. Bowen

On behalf of the other authors

----
*SUMMARY ASSESSMENT
I have now completed the evaluation of your second revised manuscript (R2) and the accompanying point-by-point response to my previous decision letter. You have made commendable progress in addressing several of the core concerns, particularly regarding:
• Explicit framing of the sample as exploratory and non-representative
• Clarification that analyses focus on pooled estimates only
• Addition of confidence intervals for Cronbach’s alpha
• Provision of complete PCA details in supplementary materials
• Revision of claims about Islamic teachings with appropriate citations
• Addition of a synthesized Conclusion section*

*However, three specific issues remain incompletely resolved from my prior decision letter. These are not fatal, but they require attention before final acceptance.*

We appreciate the positive assessment and the opportunity to further improve the manuscript.

*PERSISTING CONCERNS REQUIRING MINOR REVISION*
*1. Translation validation – limitations section remains insufficiently expanded
You correctly clarify that the survey did not include items referencing Quranic verses or principles such as Khalifah/Mizan (those emerged from open-ended responses). You also note that single-item measures preclude latent variable modeling. These clarifications are helpful.
Nevertheless, your expanded limitations text (page 27, lines 388-395) still states: “although the items were relatively straightforward and seem unlikely to vary substantially across cultural contexts.” This assumption remains methodologically unsupported. Concepts such as “climate change causes,” “willingness to promote,” and “government spending on technologies” are not self-evidently invariant across Arabic, French, Russian, Turkish, and English.
Required revision: Remove the phrase “and seem unlikely to vary substantially across cultural contexts” or replace it with a more neutral acknowledgment that cross-linguistic equivalence was not tested. The current wording downplays a genuine limitation.*

The phrase “and seem unlikely to vary substantially across cultural contexts” was removed in the last revision. As this modification was already incorporated in the revised manuscript, we believe the concern has been addressed.

*2. Mode-region confounding – sensitivity analyses still not conducted, and justification remains problematic
You decline to conduct stratified or sensitivity analyses, citing insufficient power and small subgroup cell sizes. I accept that formal subgroup comparisons are not feasible. However, you also state that “mode-related differences may have introduced additional measurement error into the pooled estimates” (page 28, lines 403-405). This is an important acknowledgment, but it is presented as a speculative limitation rather than examined in any way.
Required revision: Please add a single paragraph in the Results or Limitations section that presents the overall mean scores for key dependent variables (e.g., willingness to promote mitigation actions, perceived importance of environment) separately for paper and online respondents without claiming statistical significance or regional inference. Present these as raw descriptive values only, accompanied by a clear caveat that mode is confounded with region. This simple transparency step would allow readers to assess the potential magnitude of mode-related differences.
If you remain unwilling to provide even descriptive unadjusted comparisons, you must state explicitly that the confounding of mode with region prevents any descriptive or inferential comparison, and remove the statement that mode-related differences “may have introduced additional measurement error” (since that cannot be evaluated).*

Survey mode was substantially confounded with geographic region, such that any comparison between paper/pencil and online respondents reflects both mode and regional differences simultaneously. Consequently, even purely descriptive comparisons could be misinterpreted as evidence of mode-related effects when they cannot be disentangled from regional variation.

For this reason, we have revised the limitations section to state more explicitly that the confounding of survey mode and region prevents meaningful descriptive or inferential comparisons of potential mode effects within the current dataset. We have also removed the previous wording suggesting that mode-related differences may have introduced additional measurement error, as this possibility cannot be directly assessed with the available data.

For transparency, we now provide descriptive statistics for six study constructs by survey mode in the Supporting Information (S4 file). We have included a clear note in the S4 file that differences between online and paper respondents should not be interpreted as evidence of survey-mode effects. No statistical comparisons or substantive interpretations of these differences are presented.

*3. Theoretical framing – minor clarification still needed
You clarify that the manuscript does not name elite cue theory, moral foundations theory, or value-belief-norm theory explicitly. I have verified this: the manuscript cites empirical and theoretical work related to these frameworks without labeling them as such. That is acceptable.
However, on page 3, lines 46–55, you write: “Theoretical and empirical work suggests that individuals' climate beliefs and behavior are influenced by their morals… values… and the norms and shared beliefs of the groups with which they identify.” You then state (page 4, lines 78-82) that the study is descriptive and exploratory, not designed to test theoretical predictions.
Required revision: Add a single bridging sentence after line 82, such as: “We therefore do not test specific predictions from these theoretical frameworks but instead provide an initial empirical assessment of Muslim religious leaders’ climate-related views.” This eliminates any ambiguity about the study’s theoretical ambitions.*

We have added the following bridging sentence to the Introduction: “Accordingly, we use research suggesting the potential role of religious leaders in shaping climate-related beliefs and behaviors to contextualize the study rather than to derive formal hypotheses.” This sentence reinforces that the manuscript does not seek to test specific theoretical predictions.

We now describe our aim as follows: “This study provides descriptive, quantitative evidence on Muslim religious leaders’ beliefs about the causes and consequences of climate change, their perceptions of the role of Muslim leaders in addressing it, and their willingness to personally adopt and promote climate mitigation behaviors. Our aim is to provide an initial empirical assessment of Muslim religious leaders’ climate-related views rather than test specific theoretical predictions. Accordingly, we use research suggesting the potential role of religious leaders in shaping climate-related beliefs and behaviors to contextualize the study rather than to derive formal hypotheses.”

*ADDITIONAL MINOR ITEMS
• Abstract: The phrasing “Nearly all respondents agreed… and about half agreed…” remains slightly redundant but is acceptable. No change required.
• Conclusion: The new Conclusion section is appropriate. Well done.*

We appreciate the positive evaluation.
