# Supplementary material for: Muslim religious leaders’ views of climate change: An international survey
Source: PLoS One. 2026 Aug 25;21(8):e0353201. doi: 10.1371/journal.pone.0353201 (PMC13505925; doi:10.1371/journal.pone.0353201)
Supplement: Peer review history

# Peer review: Decision Letter 0

**DOI:** 10.1371/journal.pone.0353201.r001

**Authors:** Morufu Raimi

26 Nov 2025

Dear Dr. Bowen,

Thank you for submitting your manuscript to PLOS ONE. After careful consideration, we feel that it has merit but does not fully meet PLOS ONE’s publication criteria as it currently stands. Therefore, we invite you to submit a revised version of the manuscript that addresses the points raised during the review process.

Please submit your revised manuscript by Jan 10 2026 11:59PM. If you will need more time than this to complete your revisions, please reply to this message or contact the journal office at plosone@plos.org. When you're ready to submit your revision, log on to https://www.editorialmanager.com/pone/ and select the 'Submissions Needing Revision' folder to locate your manuscript file.

- A rebuttal letter that responds to each point raised by the academic editor and reviewer(s). You should upload this letter as a separate file labeled 'Response to Reviewers'.
- A marked-up copy of your manuscript that highlights changes made to the original version. You should upload this as a separate file labeled 'Revised Manuscript with Track Changes'.
- An unmarked version of your revised paper without tracked changes. You should upload this as a separate file labeled 'Manuscript'.

If you would like to make changes to your financial disclosure, please include your updated statement in your cover letter. Guidelines for resubmitting your figure files are available below the reviewer comments at the end of this letter.

If applicable, we recommend that you deposit your laboratory protocols in protocols.io to enhance the reproducibility of your results. Protocols.io assigns your protocol its own identifier (DOI) so that it can be cited independently in the future. For instructions see: https://journals.plos.org/plosone/s/submission-guidelines#loc-laboratory-protocols. Additionally, PLOS ONE offers an option for publishing peer-reviewed Lab Protocol articles, which describe protocols hosted on protocols.io. Read more information on sharing protocols at https://plos.org/protocols?utm_medium=editorial-email&utm_source=authorletters&utm_campaign=protocols.

We look forward to receiving your revised manuscript.

Kind regards,

Morufu Olalekan Raimi, Ph.D

Academic Editor

PLOS ONE

Journal Requirements:

When submitting your revision, we need you to address these additional requirements.

1. Please ensure that your manuscript meets PLOS ONE's style requirements, including those for file naming. The PLOS ONE style templates can be found at

https://journals.plos.org/plosone/s/file?id=wjVg/PLOSOne_formatting_sample_main_body.pdf and https://journals.plos.org/plosone/s/file?id=ba62/PLOSOne_formatting_sample_title_authors_affiliations.pdf

2. Please include a complete copy of PLOS’ questionnaire on inclusivity in global research in your revised manuscript. Our policy for research in this area aims to improve transparency in the reporting of research performed outside of researchers’ own country or community. The policy applies to researchers who have travelled to a different country to conduct research, research with Indigenous populations or their lands, and research on cultural artefacts. The questionnaire can also be requested at the journal’s discretion for any other submissions, even if these conditions are not met.  Please find more information on the policy and a link to download a blank copy of the questionnaire here: https://journals.plos.org/plosone/s/best-practices-in-research-reporting. Please upload a completed version of your questionnaire as Supporting Information when you resubmit your manuscript.

3. Please expand the acronym “CASPAR”, “KR” (as indicated in your financial disclosure) so that it states the name of your funders in full.

This information should be included in your cover letter; we will change the online submission form on your behalf.

4. We noted in your submission details that a portion of your manuscript may have been presented or published elsewhere. [A technical report of the survey is available on ResearchGate. Here, we re-frame the results for an academic audience, deepen and formalize the analysis, and include new insights and results. There is no overlap between the report and the introduction and discussion sections. New statistical elements include: a factor analysis to simply the results; the use of interquartile ranges and box plots; the use of means as indicators of central tendencies where appropriate.] Please clarify whether this publication was peer-reviewed and formally published. If this work was previously peer-reviewed and published, in the cover letter please provide the reason that this work does not constitute dual publication and should be included in the current manuscript.

If the reviewer comments include a recommendation to cite specific previously published works, please review and evaluate these publications to determine whether they are relevant and should be cited. There is no requirement to cite these works unless the editor has indicated otherwise.

Additional Editor Comments:

PLOS ONE Editorial Decision

Manuscript Number: PONE-D-25-52492

Title: “Muslim religious leaders’ views of climate change: An international survey”

Article Type: Research Article

Corresponding Author: Dr. Catherine E. Bowen

Decision: Major Revisions

Dear Dr. Bowen and Co-authors,

Thank you for submitting your manuscript to PLOS ONE. It has been evaluated by five independent reviewers, and I have now assessed it myself, along with the reviewers' comments. While the reviewers and I find the topic timely, relevant, and of potential interest to a broad readership, there are significant concerns that must be addressed before the manuscript can be considered for publication.

The study presents a valuable first international survey of Muslim religious leaders' views on climate change, a critically under-researched area. However, as noted by multiple reviewers, the manuscript in its current form requires substantial revisions to meet the methodological, analytical, and presentation standards expected by PLOS ONE.

The most critical issues that must be addressed are:

1. There are significant concerns regarding the sampling strategy, data collection procedures, and statistical reporting. A more detailed justification of the methods and enhanced transparency are required.

2. The introduction needs to more clearly articulate the specific research gap this study fills, distinguishing it from existing literature and explicitly stating its novelty.

3. The results section is currently largely descriptive. A deeper interpretation of the findings, linking them to theoretical frameworks and contextual factors, is necessary.

4. The manuscript requires thorough copyediting to improve grammar, sentence flow, and overall clarity. Figures and captions must be made self-explanatory and consistent.

A point-by-point response to all reviewer comments is required. The reviewers have provided extensive and constructive feedback, which, if addressed comprehensively, will significantly strengthen the manuscript.

Specific Required Revisions:

Please address the following points, which synthesize and prioritize the concerns raised by all reviewers:

1. Methodological Clarifications and Enhancements (Primarily from Reviewers 1, 2, & 3):

Sampling Bias: Acknowledge the limitations of the convenience and snowball sampling method more explicitly. Discuss potential biases (e.g., over-representation of climate-engaged leaders) and how they might affect the generalizability of the findings. While increasing the sample size (as suggested by Reviewer 2) is not feasible post-hoc, a more nuanced discussion of this limitation is essential.

Participant Demographics: Provide a detailed table (as suggested by Reviewer 3) summarizing the number of participants from each country, their roles (e.g., imam, teacher), school of jurisprudence, and mode of survey completion (online vs. paper).

Survey Instrument and Translation: Clarify the procedures used for translating and validating the questionnaire across multiple languages (e.g., back-translation, expert review) to ensure conceptual equivalence (Reviewer 5).

Factor Analysis: Provide a complete description of the factor analysis in the main text or the supplementary materials. This must include the extraction method (e.g., Principal Component Analysis), rotation method, Kaiser-Meyer-Olkin (KMO) measure, Bartlett's test of sphericity results, and a full table of factor loadings (Reviewer 5).

Statistical Analysis:

Justify the use of parametric tests (t-tests) on Likert-type data, citing relevant methodological literature on treating such scales as interval data (Reviewer 5).

Report confidence intervals for Cronbach's alpha values (Reviewer 3).

Conduct and report a comparative analysis between paper-based and online respondents to check for mode effects (Reviewer 3).

2. Introduction and Theoretical Framing (Primarily from Reviewers 1 & 5):

Research Gap: Sharply define the research gap in the introduction. Explicitly state how this study moves beyond existing theoretical work on Islamic environmental ethics and limited surveys of Christian leaders.

Theoretical Framework: Strengthen the theoretical underpinnings by integrating relevant frameworks such as elite cue theory, moral foundations theory, or value-belief-norm theory to better frame why religious leaders' views matter (Reviewer 5).

Literature Review: Integrate the suggested literature (e.g., Koehrsen, 2021; Mazaheri, 2024) to better contextualize the study within the emerging field of Islam and climate change.

3. Results and Discussion (Primarily from Reviewers 1, 3, & 5):

Beyond Description: The results section should not only report findings but also provide initial interpretation and highlight key patterns. Avoid simply restating what is evident in the figures and tables.

Nuanced Discussion: The discussion should more deeply explore the nuances of the findings. For example:

i. When comparing results with Christian clergy studies, discuss the distinct political, cultural, and theological contexts that may explain similarities or differences (Reviewer 5).

ii. Elaborate on the potential reasons behind the reluctance to promote dietary changes and fertility limitation, moving beyond speculation to a more critical analysis grounded in the literature on Islamic ethics and demographic debates.

iii. Policy Implications: The conclusion should be concise and include clear, actionable policy implications derived from the findings, as suggested by Reviewer 1.

4. Data and Code Availability (Primarily from Reviewer 3):

Full Data Disclosure: Ensure that the data provided via the OSF link constitutes the complete, de-identified dataset used for all analyses in the manuscript. This should include the raw data, the codebook variable definitions, and the complete SPSS (or other software) syntax scripts used for data cleaning and analysis. The current statement must be verified and confirmed.

5. Language and Presentation (All Reviewers):

Professional Copyediting: The manuscript must undergo thorough professional English language editing to correct grammatical errors, improve sentence construction, eliminate redundancy, and enhance overall readability.

Figures and Captions: Ensure all figures are high-resolution and that all captions are uniform, self-explanatory, and explicitly mention the sample size (N=150). Each axis must be clearly labeled, and the caption should fully describe what the figure depicts without requiring the reader to refer back to the main text (Reviewers 1 & 3).

We hope that you will find the reviewers' comments helpful as you revise your manuscript. Please submit a revised version that addresses all the points raised, along with a detailed point-by-point response to the reviewers.

Please note that your revised manuscript will be re-evaluated, and acceptance is not guaranteed.

Thank you for considering PLOS ONE as a venue for your work.

Sincerely,

Prof. Morufu Olalekan Raimi

Academic Editor, PLOS ONE

[Note: HTML markup is below. Please do not edit.]

Reviewers' comments:

Reviewer's Responses to Questions

**Comments to the Author**

1. Is the manuscript technically sound, and do the data support the conclusions?

Reviewer #1: Yes

Reviewer #2: Yes

Reviewer #3: Partly

Reviewer #4: Yes

Reviewer #5: Yes

**********

2. Has the statistical analysis been performed appropriately and rigorously?

Reviewer #1: Yes

Reviewer #2: Yes

Reviewer #3: No

Reviewer #4: Yes

Reviewer #5: Yes

**********

3. Have the authors made all data underlying the findings in their manuscript fully available?

The PLOS Data policy

Reviewer #1: Yes

Reviewer #2: Yes

Reviewer #3: No

Reviewer #4: Yes

Reviewer #5: Yes

**********

4. Is the manuscript presented in an intelligible fashion and written in standard English?

Reviewer #1: Yes

Reviewer #2: Yes

Reviewer #3: Yes

Reviewer #4: Yes

Reviewer #5: Yes

**********

Reviewer #1: ARTICLE TITLE: Muslim religious leaders’ views of climate change: An international survey

Significance and Novelty: (a) This study provides one of the first global assessments of how Muslim religious leaders understand and interpret climate change, offering insights that are both socially and academically important. Religious leaders hold significant influence within Muslim communities, shaping public attitudes, guiding behavioral norms, and informing environmental ethics grounded in Islamic teachings.

(b) By capturing perspectives from diverse countries, the survey highlights the extent to which climate change is recognized as a moral, spiritual, and social concern within Islamic discourse.

(c) The study’s novelty lies in its international scope and its focus on a population rarely examined in climate-perception research. It reveals variations and commonalities in religious framing, perceived responsibilities, and willingness to support climate action.

(d) Ultimately, this work expands the conversation on climate change by integrating faith-based viewpoints, providing invaluable insights for policymakers, sustainability practitioners, and scholars seeking to engage religious communities in global environmental efforts.

Decision: Minor Revisions

1. Although the manuscript integrates various known techniques, it does not provide any novel framework, methodology, or insight. The combination of known tools without methodological innovation or novel interpretation fails to meet the standard for publication in a high-impact journal. (Improve by incorporating relevant studies in LR section)

2. The manuscript suffers from severe grammatical errors, awkward sentence constructions, redundancy, and poor flow. The text is often difficult to follow, diminishing the clarity and professionalism required for scholarly communication.

3. Ensure figure captions are uniform and self-explanatory.

4. The manuscript lacks a clear research gap statement and fails to highlight how this work differs from previous studies in India or elsewhere. The novelty should be explicitly clarified in the Introduction.

5. Results Interpretation: The Results section reads descriptively with little interpretation or linkage to IMPLEMENTED processes.

6. The conclusion should be concise and highlight the key findings and policy implications.

7. Numerous references are cited in a generic manner without clear relevance to the specific arguments or methods used. This suggests inadequate critical engagement with existing literature.

The manuscript, while addressing an important topic, falls significantly short in terms of scientific rigor, methodological appropriateness, language quality, and originality. The issues are foundational and cannot be addressed with minor revisions.

Various articles for improvement

1. Koehrsen, J. (2021). Muslims and climate change: How Islam, Muslim organizations, and religious leaders influence climate change perceptions and mitigation activities. Wiley Interdisciplinary Reviews: Climate Change, 12(3), e702.

2. Skirbekk, V., & Pędziwiatr, K. (2018). Sustainability and climate change in major religions with a focus on Islam. Humanitarian Academy for World Development Research Paper.

3. Mazaheri, N. (2024). Faith in science: religion and climate change attitudes in the Middle East. Global Environmental Politics, 24(1), 52-75.

4. Ali, A., Ali, S., Baird, I. G., & Yaseen, M. (2025). Exploring the potential role of Muslim spiritual leaders in coping with climate change through tree planting in Pakistan. Local Environment, 30(4), 520-533.

5. Agusalim, L., & Karim, M. (2024). Religiosity and climate change: An eco-religious approach. Environmental & Socio-Economic Studies, 12(1), 35-50.

Reviewer #2: It would have been great. If the author could have further increased the weightage of the paper by increasing the sample size from 150 to 1500 or as maximum as possible. However the manuscript is accepted in the current form.

Reviewer #3: Although the study addresses an important issue, it has several fundamental methodological shortcomings. There is a risk of bias due to the use of possessive and snowball sampling, the number of people invited, and how many responded. Statistical analysis appears to be performed to maintain general standards, but important information is missing. The full procedure for factor analysis is not described.

No confidence intervals were added for Cronbach's alpha, and no potential differences were analyzed between paper-based and online participants, which were very important.

The authors have not disclosed the complete data in this study. The authors mention the availability of OSF data and SPSS, but the link provided cannot be confirmed as to whether the data set is complete or not. The Raw data, court book, and complete analysis scripts need to be released for re-evaluation. The data currently provided is not up to standard.

The research is fairly understandable, but there is Scope for improvement. The language used in discussions of religion and demographics should be both neutral and sensitive. The caption of each figure is not clear; the axis name, scale, and sample size should be mentioned. Find this study incomplete because there are some missing questions. However, with some corrections, this study can be made readable.

Additional comments-

1. Around 150 Muslim religious leaders’ Precipitant and give their feedback, but it is totally Unclear (Need more information about them). You need to add the total number of participants from each country to a separate table.

2. Any differences between paper and online participants should be analyzed and noted.

3. You need to add a complete copy of the questionnaire, and it should be attached.

4.It would be very helpful for readers and the researcher if the language were more sensitive and neutral in discussions of religion and demographics.

Reviewer #4: The manuscript obtained data through a questionnaire survey, described a technically reasonable scientific research, and provided data to support the conclusion. For instance, the author presented through us the results of a global survey of Muslim religious leaders (N=150) from 29 countries, such as Imams, religious teachers, and students of religious colleges. Our research findings indicate that the majority of respondents believe that climate change is man-made and that it has already caused serious harm. Participants also highly agreed that the Muslim religious community should make more efforts to address climate change. Islam provides a set of values and principles that encourage environmentally responsible behavior. Respondents reported that they sometimes involved climate change and other environmental issues in their religious work. They expressed their strong willingness to encourage the government to invest in technologies aimed at curbing emissions and the impacts of climate change. Adopt new technologies to promote more environmentally friendly production and consumption; Reduced consumption. There is a relatively low willingness to promote adjustments in dietary habits and reduce meat consumption. Vegetarianism Or limit population growth and family size. The logic is clear and the conclusion is definite. It is an article worth discussing. At the same time, appropriate and strict statistical analysis was carried out. In addition to summary statistics, data points behind mean, median and variance measures should also be provided. The writing of the manuscript is also relatively easy to understand, and the English writing is also quite standardized.

Reviewer #5: This paper discusses the important issue of climate-change attitudes among Muslim religious leaders globally. The Paper has broader reader interest and may be published after addressing the following issues:

1. Page 1 Lines 18–31 (Abstract) The abstract is clear but overly descriptive. The abstract provides a theoretical explanation for the significance of Muslim religious leaders, citing factors such as moral authority and social capital.

2. Page 2 Lines 33–44 The introduction states the importance of religious leaders but could benefit from clearer theoretical framework (e.g., moral foundations, social identity, and elite cue theory).

3. Page 3 Line 41 The introduction states the importance of religious leaders but could benefit from clearer theoretical framework (e.g., moral foundations, social identity, and elite cue theory).

4. Page 3 Line 42 “wary of science…in particular.” This is accurate but needs nuance to avoid suggesting religious people are uniformly anti-science.

5. Page 3 Line 66 representing why Muslim leaders are uniquely understudied.

6. Page 4-5 line 101-105 Useful but explain how this relates to your analyses. Did you test urban/rural differences? If not, move this to descriptive table

7. Page 6 Lines 110–113 Because the questionnaire was administered in multiple languages, please clarify whether translations were checked (e.g., via back-translation or expert review). This ensures consistency across survey modes.

8. Page 5 lines 118–133 Only minimal information is provided. Please add the extraction method (e.g., principal components or maximum likelihood) and KMO measure and Bartlett’s test

9. Page 6 line 148-150 statistical methods are straightforward, but justify why parametric tests were used with 11-point Likert items, but still useful to mention its robustness

10. Page 9 Lines 263-271 Avoid overstating consistency with Christian studies; Muslim contexts differ culturally and politically. Add nuance: differences in democratic participation, political risks, etc.

**********

what does this mean?). If published, this will include your full peer review and any attached files.

If you choose “no”, your identity will remain anonymous but your review may still be made public.

**Do you want your identity to be public for this peer review?**  For information about this choice, including consent withdrawal, please see our Privacy Policy

Reviewer #1: No

Reviewer #2: **Yes:**  Taimoor Shah Durrani

Reviewer #3: **Yes:**  Md Nazmul Hossain

Reviewer #4: No

Reviewer #5: **Yes:**  Abdullah Al Mamun

**********

[NOTE: If reviewer comments were submitted as an attachment file, they will be attached to this email and accessible via the submission site. Please log into your account, locate the manuscript record, and check for the action link "View Attachments". If this link does not appear, there are no attachment files.]

To ensure your figures meet our technical requirements, please review our figure guidelines: https://journals.plos.org/plosone/s/figures

You may also use PLOS’s free figure tool, NAAS, to help you prepare publication quality figures: https://journals.plos.org/plosone/s/figures#loc-tools-for-figure-preparation.

NAAS will assess whether your figures meet our technical requirements by comparing each figure against our figure specifications.

Attachment Submitted filename: Remarks to the Author.docx

Attachment Submitted filename: 123.docx

# Author comment: Author response to Decision Letter 1

**DOI:** 10.1371/journal.pone.0353201.r002

13 Feb 2026

Response to Editor and Reviewers [PONE-D-25-52492]

Authors: Following consultation with the PLOS ONE editorial staff (Case No. 09388620), we were advised that a response to Reviewer 1’s comments was not required. We have therefore focused our efforts on addressing the remaining editor and reviewer comments.

_______________________________

Editor

The most critical issues that must be addressed are:

1. There are significant concerns regarding the sampling strategy, data collection procedures, and statistical reporting. A more detailed justification of the methods and enhanced transparency are required.

Authors: We have addressed the reviewers’ concerns in the point-by-point rebuttal below. Briefly, we have elaborated on the limitations of our sampling strategy and described some additional limitations; provided information about the translation procedure; and provided more information about the statistical approach. The questionnaire, raw data, and SPSS data analysis scripts are available on the Open Science Foundation (OSF) repository. Below, line numbers refer to the version of the revised manuscript with Track Changes.

2. The introduction needs to more clearly articulate the specific research gap this study fills, distinguishing it from existing literature and explicitly stating its novelty.

Authors: We have re-written the Introduction to make the aim and novelty of the study more explicit. We now write on lines 78 -83: In the current study, we provide quantitative evidence on Muslim religious leaders’ beliefs about the causes and consequences of climate change, their perceptions of the role of Muslim leaders in addressing climate change, and their willingness to personally engage in and promote climate mitigation behaviors. Few studies have examined religious leaders’ climate views, and most existing research has been either qualitative or focused primarily on Christian leaders. By analyzing survey data from Muslim religious leaders, this study addresses an important gap in the literature.

3. The results section is currently largely descriptive. A deeper interpretation of the findings, linking them to theoretical frameworks and contextual factors, is necessary.

Authors: We have aimed to adhere to PLOS ONE guidelines. According these guidelines, authors may choose to present their results and discussion either separately or in a combined “Results and Discussion” section. We have chosen to present them separately, with the Results section focusing on objective presentation of the findings and interpretation provided in the Discussion section. To ensure this distinction is clear, we have reviewed the manuscript and strengthened the interpretive components in the Discussion where appropriate.

4. The manuscript requires thorough copyediting to improve grammar, sentence flow, and overall clarity. Figures and captions must be made self-explanatory and consistent.

Authors: We have thoroughly proofread the manuscript and revised several passages.

Specific Required Revisions:

Please address the following points, which synthesize and prioritize the concerns raised by all reviewers:

1. Methodological Clarifications and Enhancements (Primarily from Reviewers 1, 2, & 3):

Sampling Bias: Acknowledge the limitations of the convenience and snowball sampling method more explicitly. Discuss potential biases (e.g., over-representation of climate-engaged leaders) and how they might affect the generalizability of the findings. While increasing the sample size (as suggested by Reviewer 2) is not feasible post-hoc, a more nuanced discussion of this limitation is essential.

Authors: We have now elaborated on the limitations of our sampling method, sample size, and potential biases in the Strengths, limitations, and future research section (lines 371-395).

Participant Demographics: Provide a detailed table (as suggested by Reviewer 3) summarizing the number of participants from each country, their roles (e.g., imam, teacher), school of jurisprudence, and mode of survey completion (online vs. paper).

Authors: We have now moved the descriptive statistics from the main text into a dedicated table (Table 1), which summarizes the number of participants by country, their roles (e.g., imam, teacher), school of jurisprudence, and mode of survey completion (online vs. paper). The table also includes additional relevant demographic characteristics for completeness.

Survey Instrument and Translation: Clarify the procedures used for translating and validating the questionnaire across multiple languages (e.g., back-translation, expert review) to ensure conceptual equivalence (Reviewer 5).

Authors: We have now clarified the translation procedure in the Methods/Procedure section (lines 118-122). While we did not conduct formal statistical tests of measurement equivalence across language versions, the items consisted primarily of direct and concrete questions rather than culturally-nuanced or complex constructs. Nevertheless, we recognize that the absence of formal equivalence testing is a limitation and have now acknowledged this explicitly in the manuscript (lines 388-390).

Factor Analysis: Provide a complete description of the factor analysis in the main text or the supplementary materials. This must include the extraction method (e.g., Principal Component Analysis), rotation method, Kaiser-Meyer-Olkin (KMO) measure, Bartlett's test of sphericity results, and a full table of factor loadings (Reviewer 5).

Authors: In the main text, we have clarified that we conducted a principal components analysis (PCA) with orthogonal varimax rotation and Kaiser normalization (lines 146-147). Further details and the complete results of the analysis - including the extraction method, rotation method, KMO measure, Bartlett’s test of sphericity, and the full table of component loadings - are provided in the S2 file. To ensure clarity, we have made the reference to the S2 file more explicit (line 154-155).

Statistical Analysis: Justify the use of parametric tests (t-tests) on Likert-type data, citing relevant methodological literature on treating such scales as interval data (Reviewer 5).

Authors: The manuscript includes a citation to methodological literature supporting the use of parametric tests with 11-point Likert-type scales; we have revised the text to make this justification more explicit (lines 179-180).

Report confidence intervals for Cronbach's alpha values (Reviewer 3).

Authors: We have added 95% confidence interval for the Cronbach's alpha values on (lines 156-158 and 166). We have likewise amended the SPSS script on the OSF repository.

Conduct and report a comparative analysis between paper-based and online respondents to check for mode effects (Reviewer 3).

Authors: We employed a mixed-mode design (paper-based and online questionnaires) to maximize coverage and accessibility across regions and linguistic groups. Mixed-mode approaches can enhance inclusivity and improve reach, particularly when sampling geographically dispersed or hard-to-access populations.

However, in our sample, mode of data collection is significantly associated with region of residence (χ²(4, N = 149) = 38.37, p < .001; see cross-tabulation below). This creates a structural confound between survey mode and region. Consequently, any observed differences between paper-based and online respondents could reflect regional variation rather than mode effects per se, rendering direct comparisons ambiguous.

Moreover, subsample sizes within regions are insufficient for adequately powered within-region mode comparisons. For example, detecting a medium effect (Cohen’s d = 0.5) with α = .10 and 80% power would require approximately 51 participants per mode within each region, which is not met in the present data. Within several regions, mode is additionally confounded with country of residence.

For these reasons, we do not consider a comparative analysis between modes to yield interpretable results. Nevertheless, we agree with the reviewers that survey mode may have influenced responses. We have now explicitly noted this as a limitation in the Strengths, limitations, and future research section (lines 390-395).

Paper/pencil or online questionnaire * world region of residence Crosstabulation

Count

world region of residence Total

Western Europe and USA post-Soviet MENA sub-Saharan Africa Asia

paper/pencil or online questionnaire paper/pencil 15 0 13 22 20 70

online 17 17 31 6 8 79

Total 32 17 44 28 28 149

2. Introduction and Theoretical Framing (Primarily from Reviewers 1 & 5):

Research Gap: Sharply define the research gap in the introduction. Explicitly state how this study moves beyond existing theoretical work on Islamic environmental ethics and limited surveys of Christian leaders.

Authors: We agree with the reviewers that the research gap and the study’s contribution needed to be more sharply articulated. We have therefore revised the Introduction to make the aim and novelty of the study more explicit. We now write on lines 78 -83: In the current study, we provide quantitative evidence on Muslim religious leaders’ beliefs about the causes and consequences of climate change, their perceptions of the role of Muslim leaders in addressing climate change, and their willingness to personally engage in and promote climate mitigation behaviors. Few studies have examined religious leaders’ climate views, and most existing research has been either qualitative or focused primarily on Christian leaders. By analyzing survey data from Muslim religious leaders, this study addresses an important gap in the literature.

Theoretical Framework: Strengthen the theoretical underpinnings by integrating relevant frameworks such as elite cue theory, moral foundations theory, or value-belief-norm theory to better frame why religious leaders' views matter (Reviewer 5).

Authors: We agree with the reviewer that the theoretical underpinnings of the study could be strengthened. In response, we have revised the first paragraph of the Introduction to explicitly reference theoretical and empirical work on the role of elite cues, moral values, and social identities in shaping climate beliefs and behaviors, thereby reinforcing why religious leaders’ views are important. We have also added seven new references to support this framing.

Literature Review: Integrate the suggested literature (e.g., Koehrsen, 2021; Mazaheri, 2024) to better contextualize the study within the emerging field of Islam and climate change.

Authors: We appreciate the literature suggestions. In response, we have added a reference to Mazaheri (2024) in the Discussion section (reference #30, line 288). Koehrsen (2021) was already cited in the manuscript (reference #34; lines 320 and 366).

3. Results and Discussion (Primarily from Reviewers 1, 3, & 5):

Beyond Description: The results section should not only report findings but also provide initial interpretation and highlight key patterns. Avoid simply restating what is evident in the figures and tables.

Authors: We aimed to adhere to PLOS ONE guidelines, which state that authors may choose to present their results and discussion either separately or in a combined “Results and Discussion” section. We have chosen to present them separately, with the Results section focusing on objective presentation of the findings and interpretation provided in the Discussion section. To ensure this distinction is clear, we have reviewed the manuscript and strengthened the interpretive components in the Discussion where appropriate.

Nuanced Discussion: The discussion should more deeply explore the nuances of the findings. For example:

i. When comparing results with Christian clergy studies, discuss the distinct political, cultural, and theological contexts that may explain similarities or differences (Reviewer 5).

Authors: We appreciate the suggestion to discuss what might explain why both the Muslim leaders in our study, and the (mostly) Christian leaders in the study from Syropoulos and Sparkman acknowledge human-caused climate change but do not meaningfully address it in their work as religious leaders. We now write (lines 306-315): The observation that both Muslim leaders in the current study and primarily Christian leaders in the study from Syropoulos and Sparkman (14) acknowledge anthropogenic climate change yet rarely address it in their religious work suggests convergence in behavior, but not necessarily the reasons for it. Some reasons may cut across faith groups, such as deprioritizing climate change in the face of more immediate concerns (providing moral or spiritual guidance; supporting community members during crises), or having limited guidance on how to meaningfully integrate environmental issues into their religious work. Other reasons may be specific to religious leaders of particular faiths or in specific social contexts. Future research should examine why religious leaders do not address climate change in their religious work, recognizing that the reasons may potentially vary across theological traditions, institutional structures, political contexts, and geographies.

ii. Elaborate on the potential reasons behind the reluctance to promote dietary changes and fertility limitation, moving beyond speculation to a more critical analysis grounded in the literature on Islamic ethics and demographic debates.

Authors: We appreciate the reviewer’s suggestion to elaborate on potential reasons behind respondents’ reluctance to promote dietary change and fertility limitation. Our study did not collect data on these underlying motivations, so any discussion would necessarily be speculative and rely on existing literature. Additionally, we do not have specialized training in Islamic jurisprudence or ethical theory; providing a detailed theological or jurisprudential analysis would fall outside the appropriate scope of this manuscript and could risk overstating our expertise.

In response to the reviewer’s suggestions, we now highlight the need for future research that integrates empirical investigation with a critical engagement with the literature on Islamic ethics to better understand why Muslim religious leaders may be reluctant to promote dietary change and fertility limitation as strategies for mitigating climate change (lines 402-405).

iii. Policy Implications: The conclusion should be concise and include clear, actionable policy implications derived from the findings, as suggested by Reviewer 1.

Authors: We have not addressed Reviewer 1’s comments in this revision following consultation with the PLOS ONE editorial staff (Case No. 09388620).

We note that our findings are based on a convenience sample of N=150 Muslim religious leaders from diverse cultural and social contexts. Given the modest sample size and substantial heterogeneity across contexts, deriving specific, actionable policy recommendations would overextend the data. We do, however, suggest that interventions aimed at enhancing Muslim religious leaders’ self-efficacy to engage in political campaigns may help unlock their potential to motivate climate-friendly policies and community-wide action (lines 336-338). In the Strengths, limitations, and future research section, we now explicitly note that the non-representative and heterogeneous nature of our sample precludes generalizable policy recommendations (lines 382-384).

4. Data and Code Availability (Primarily from Reviewer 3):

Full Data Disclosure: Ensure that the data provided via the OSF link constitutes the complete, de-identified dataset used for all analyses in the manuscript. This should include the raw data, the codebook variable definitions, and the complete SPSS (or other software) syntax scripts used for data cleaning and analysis. The current statement must be verified and confirmed.

Authors: We appreciate the importance of transparency. The OSF repository includes the raw data, the complete SPSS syntax scripts, a codebook with variable definitions, and the questionnaire. The wording of all questionnaire items analyzed in the current study remains available in the S1 file. We confirm that the OSF repository contains the complete, de-identified dataset used for all analyses reported in the manus

Attachment Submitted filename: Response to Reviewers.docx

# Editor report: Decision Letter 1

**DOI:** 10.1371/journal.pone.0353201.r003

**Authors:** Morufu Raimi

27 Mar 2026

Dear Dr. Bowen,

Thank you for submitting your manuscript to PLOS ONE. After careful consideration, we feel that it has merit but does not fully meet PLOS ONE’s publication criteria as it currently stands. Therefore, we invite you to submit a revised version of the manuscript that addresses the points raised during the review process.

Please submit your revised manuscript by May 11 2026 11:59PM. If you will need more time than this to complete your revisions, please reply to this message or contact the journal office at plosone@plos.org. When you're ready to submit your revision, log on to https://www.editorialmanager.com/pone/ and select the 'Submissions Needing Revision' folder to locate your manuscript file.

- A letter that responds to each point raised by the academic editor and reviewer(s). You should upload this letter as a separate file labeled 'Response to Reviewers'.
- A marked-up copy of your manuscript that highlights changes made to the original version. You should upload this as a separate file labeled 'Revised Manuscript with Track Changes'.
- An unmarked version of your revised paper without tracked changes. You should upload this as a separate file labeled 'Manuscript'.

If applicable, we recommend that you deposit your laboratory protocols in protocols.io to enhance the reproducibility of your results. Protocols.io assigns your protocol its own identifier (DOI) so that it can be cited independently in the future. For instructions see: https://journals.plos.org/plosone/s/submission-guidelines#loc-laboratory-protocols. Additionally, PLOS ONE offers an option for publishing peer-reviewed Lab Protocol articles, which describe protocols hosted on protocols.io. Read more information on sharing protocols at https://plos.org/protocols?utm_medium=editorial-email&utm_source=authorletters&utm_campaign=protocols.

We look forward to receiving your revised manuscript.

Kind regards,

Morufu Olalekan Raimi, Ph.D

Academic Editor

PLOS One

Journal Requirements:

If the reviewer comments include a recommendation to cite specific previously published works, please review and evaluate these publications to determine whether they are relevant and should be cited. There is no requirement to cite these works unless the editor has indicated otherwise.

Please review your reference list to ensure that it is complete and correct. If you have cited papers that have been retracted, please include the rationale for doing so in the manuscript text, or remove these references and replace them with relevant current references. Any changes to the reference list should be mentioned in the rebuttal letter that accompanies your revised manuscript. If you need to cite a retracted article, indicate the article’s retracted status in the References list and also include a citation and full reference for the retraction notice.

Additional Editor Comments:

PLOS ONE Editorial Decision

Manuscript Number: PONE-D-25-52492R1

Title: Muslim religious leaders' views of climate change: An international survey

Authors: Catherine E. Bowen, Konrad Pedziwiatr, Marcin Stonawski, Vegard Skirbekk

Dear Dr. Bowen and Co-authors,

Thank you for submitting your revised manuscript to PLOS ONE and for your detailed response to the previous round of reviews. I have carefully examined the revised version, the comprehensive point-by-point response, and the cumulative feedback from five reviewers across two review cycles. The authors have demonstrated genuine effort in addressing the methodological and theoretical concerns raised. The addition of a detailed demographic table (Table 1), clarification of the translation procedures, expanded discussion of sampling limitations, and strengthened theoretical framing in the introduction represent meaningful improvements. The transparency enhancements, including confidence intervals for Cronbach's alpha, complete PCA details in supplementary materials, and full data availability via OSF, are commendable and align with PLOS ONE's standards. However, after two revision cycles, several substantive concerns remain insufficiently addressed, and new issues have emerged that require attention before this manuscript can be considered for publication.

Persistent and Emerging Concerns Requiring Resolution

1. The Sampling Limitation Acknowledgment Remains Inadequate

The authors now acknowledge that their convenience and snowball sample “may not be representative of Muslim leaders worldwide” and note that “snowball sampling can introduce bias.” However, the magnitude and direction of potential bias are not sufficiently characterized.

With 150 respondents from 29 countries, averaging approximately 5 respondents per country, with 12 countries represented by a single individual, the sample cannot support any claims about cross-national patterns or even provide meaningful descriptive data for most countries represented. The United Kingdom alone accounts for 13.3% of the sample (20 respondents), while vast regions such as sub-Saharan Africa (28 respondents across 6 countries) and Asia (28 respondents across 5 countries) are aggregated in ways that obscure enormous cultural, political, and theological diversity.

Required: The authors must explicitly state that this sample provides exploratory insights rather than representative data, and should refrain from any language suggesting cross-regional comparisons or generalizable findings. The current phrasing "first impression" is appropriate but should be emphasized more prominently in the abstract and conclusion.

2. The Translation Validation Issue Is Not Resolved

The authors now clarify that “no back-translation or formal validation procedures were employed” and that English served as the master version for forward-translation into Arabic, French, Russian, and Turkish. They acknowledge this as a limitation, stating that items were “relatively straightforward and seem unlikely to vary substantially across cultural contexts.”

This defense is problematic for three reasons:

First, concepts such as “climate change,” “environmental responsibility,” and “political efficacy” are not straightforward across cultures; they are precisely the kinds of constructs that require rigorous cross-cultural validation. The assumption that these translate equivalently without back-translation is methodologically unsupported.

Second, the items include references to specific Quranic verses and Islamic principles (Khalifah, Mizan) that were presented in translation. The equivalence of religious terminology across languages is particularly sensitive and requires expert review.

Third, the survey was administered in five languages to respondents across 29 countries, yet no empirical demonstration of measurement equivalence is provided. The absence of formal validation undermines confidence that respondents across linguistic groups interpreted items comparably.

Required: Either (a) conduct a post-hoc analysis demonstrating measurement equivalence across language versions (e.g., multi-group confirmatory factor analysis) if sample sizes permit, or (b) substantially expand the limitations section to explicitly state that cross-linguistic comparability cannot be assured, and remove any claims that implicitly assume such comparability.

3. The Mode Effects Analysis Remains Problematic

The authors correctly note that survey mode is confounded with region, precluding direct comparison between paper-based and online respondents. However, their response raises a more fundamental concern: the sampling strategy produced a dataset in which mode and region are inseparable. This is not merely a limitation to be acknowledged; it reflects a design flaw that fundamentally compromises the interpretability of any findings that might vary by region or mode.

The cross-tabulation provided reveals that:

• 100% of post-Soviet respondents completed online surveys

• 100% of Western European/US respondents completed online surveys

• 71% of MENA respondents completed paper surveys

• 79% of sub-Saharan African respondents completed paper surveys

This pattern suggests systematic differences in how data were collected across regions, potentially introducing unmeasured variance that cannot be statistically controlled. The authors' decision to “acknowledge” rather than address this is insufficient.

Required: The authors must conduct sensitivity analyses examining whether key findings (e.g., willingness to promote specific mitigation actions) differ when stratified by region, even if mode comparisons are not possible. If regional patterns emerge that could plausibly reflect mode effects rather than true regional differences, this must be explicitly discussed as a threat to validity.

4. The Theoretical Framing, While Improved, Remains Superficial

The authors have added references to elite cue theory, moral foundations theory, and value-belief-norm theory in the introduction. However, these theories are named but not integrated into the analysis or discussion. The results are not interpreted through these theoretical lenses, and the discussion does not return to them to explain findings.

For example:

• If elite cues matter, do the findings suggest that Muslim religious leaders see themselves as effective cue-givers? The data show they rarely address climate change. Does this reflect a failure of elite communication?

• If moral foundations shape environmental attitudes, how do the Islamic principles cited (Khalifah, Mizan) map onto moral foundations? This connection is not explored.

• If value-belief-norm theory explains behavior, do the data support the theory's predictions about the relationship between values, beliefs about consequences, and behavioral intentions?

Required: The authors must either (a) explicitly use these theoretical frameworks to interpret their findings in the discussion, or (b) remove the theoretical references and present the study as descriptive/exploratory without claiming theoretical grounding. The current approach, name-dropping theories without application, does not strengthen the manuscript.

5. The Discussion of Dietary Change and Fertility Limitation Remains Speculative

The authors declined to elaborate on reasons for reluctance to promote dietary change and fertility limitation, citing lack of data on underlying motivations and lack of specialized training in Islamic ethics. This response is reasonable but leaves the discussion thin.

However, the authors then state: “Islam neither encourages nor forbids vegetarianism” and “most Muslim scholars agree that Islam does not prohibit fertility regulation.” These are empirical claims about Islamic jurisprudence that require citation and justification. Without supporting references, these statements are unsupported assertions.

Required: Either (a) provide scholarly citations supporting these claims about Islamic teachings, or (b) remove these statements and focus the discussion on the empirical finding of reluctance without speculating about theological justification.

6. The Conclusion Lacks Actionable Implications

The authors declined to provide policy implications, arguing that the non-representative sample precludes generalizable recommendations. This is methodologically sound but leaves the conclusion underwhelming. The manuscript ends with a call for future research, appropriate for a limitations section, but insufficient for a conclusion.

Required: The conclusion should synthesize what this exploratory study does contribute: it demonstrates that Muslim religious leaders across diverse contexts acknowledge climate change, see Islamic values as relevant, and are willing to support certain mitigation actions. It identifies specific areas of reluctance (diet, fertility) that warrant further investigation. It provides a foundation for hypothesis generation and instrument development. The authors should frame these as contributions without overclaiming generalizability.

7. Language and Presentation Concerns Persist

Despite the authors' assertion that readability concerns were raised only by Reviewer 1, multiple reviewers noted language issues. Reviewer 1 explicitly stated: “The manuscript suffers from severe grammatical errors, awkward sentence constructions, redundancy, and poor flow.” Reviewer 3 noted “scope for improvement” and requested more sensitive language. Reviewer 5 requested clarification on multiple passages.

The revised manuscript still contains passages that require editing. For example, the abstract includes redundant phrasing: “Nearly all respondents agree... and about half agree...” The results section includes sentences that simply restate figure contents without synthesis.

Required: The manuscript requires professional copyediting by a native English speaker or professional service before resubmission. The authors should provide documentation of editing if they use a service.

Summary of Required Revisions

Before this manuscript can be considered further, the authors must:

1. Reframe the sampling limitations to explicitly characterize the sample as exploratory and non-representative, emphasizing the inability to make cross-regional comparisons or generalizable claims.

2. Address translation validity by either demonstrating measurement equivalence across language versions or substantially expanding the limitations section to acknowledge that cross-linguistic comparability cannot be assured.

3. Conduct sensitivity analyses examining whether key findings vary by region, and discuss the potential confounding of region and mode as a threat to validity.

4. Integrate the theoretical framework into the analysis and discussion, or remove theoretical references and present the study as descriptive.

5. Provide citations for claims about Islamic teachings regarding vegetarianism and fertility regulation, or remove unsupported assertions.

6. Reframe the conclusion to articulate the study's actual contributions without overclaiming generalizability.

7. Undergo professional copyediting to address language and presentation concerns.

Decision Moderate Revision Required

Given the cumulative revision history and the persistence of these substantive concerns, I cannot recommend acceptance at this time. However, the manuscript addresses an important and under-researched topic, and the authors have demonstrated commitment to improvement. The authors may choose to resubmit a substantially revised manuscript addressing the concerns outlined above. Any resubmission will be evaluated and will likely be sent for additional peer review. If the authors believe these concerns cannot be adequately addressed, they may wish to consider alternative venues better suited to exploratory, descriptive research.

Thank you for your continued engagement with the peer review process. I appreciate the scholarly effort invested in this work.

Sincerely,

Morufu Olalekan Raimi, PhD

Academic Editor

PLOS ONE

**********

[NOTE: If reviewer comments were submitted as an attachment file, they will be attached to this email and accessible via the submission site. Please log into your account, locate the manuscript record, and check for the action link "View Attachments". If this link does not appear, there are no attachment files.]

To ensure your figures meet our technical requirements, please review our figure guidelines: https://journals.plos.org/plosone/s/figures

You may also use PLOS’s free figure tool, NAAS, to help you prepare publication quality figures: https://journals.plos.org/plosone/s/figures#loc-tools-for-figure-preparation.

NAAS will assess whether your figures meet our technical requirements by comparing each figure against our figure specifications.

# Author comment: Author response to Decision Letter 2

**DOI:** 10.1371/journal.pone.0353201.r004

21 May 2026

Please see the attached document.

Attachment Submitted filename: academic editor cover letter R2.pdf

# Editor report: Decision Letter 2

**DOI:** 10.1371/journal.pone.0353201.r005

**Authors:** Morufu Raimi

29 May 2026

Dear Dr. Bowen,

Thank you for submitting your manuscript to PLOS ONE. After careful consideration, we feel that it has merit but does not fully meet PLOS ONE’s publication criteria as it currently stands. Therefore, we invite you to submit a revised version of the manuscript that addresses the points raised during the review process.

Please submit your revised manuscript by Jul 13 2026 11:59PM. If you will need more time than this to complete your revisions, please reply to this message or contact the journal office at plosone@plos.org. When you're ready to submit your revision, log on to https://www.editorialmanager.com/pone/ and select the 'Submissions Needing Revision' folder to locate your manuscript file.

- A letter that responds to each point raised by the academic editor and reviewer(s). You should upload this letter as a separate file labeled 'Response to Reviewers'.
- A marked-up copy of your manuscript that highlights changes made to the original version. You should upload this as a separate file labeled 'Revised Manuscript with Track Changes'.
- An unmarked version of your revised paper without tracked changes. You should upload this as a separate file labeled 'Manuscript'.

If you would like to make changes to your financial disclosure, please include your updated statement in your cover letter. Guidelines for resubmitting your figure files are available below the reviewer comments at the end of this letter.

If applicable, we recommend that you deposit your laboratory protocols in protocols.io to enhance the reproducibility of your results. Protocols.io assigns your protocol its own identifier (DOI) so that it can be cited independently in the future. For instructions see: https://journals.plos.org/plosone/s/submission-guidelines#loc-laboratory-protocols. Additionally, PLOS ONE offers an option for publishing peer-reviewed Lab Protocol articles, which describe protocols hosted on protocols.io. Read more information on sharing protocols at https://plos.org/protocols?utm_medium=editorial-email&utm_source=authorletters&utm_campaign=protocols.

As the corresponding author, your ORCID iD is verified in the submission system and will appear in the published article. PLOS supports the use of ORCID, and we encourage all coauthors to register for an ORCID iD and use it as well. Please encourage your coauthors to verify their ORCID iD within the submission system before final acceptance, as unverified ORCID iDs will not appear in the published article. *Only*  the individual author can complete the verification step; PLOS staff *cannot*  verify ORCID iDs on behalf of authors.

We look forward to receiving your revised manuscript.

Kind regards,

Morufu Olalekan Raimi, Ph.D

Academic Editor

PLOS One

Journal Requirements:

1. If the reviewer comments include a recommendation to cite specific previously published works, please review and evaluate these publications to determine whether they are relevant and should be cited. There is no requirement to cite these works unless the editor has indicated otherwise.

2. Please review your reference list to ensure that it is complete and correct. If you have cited papers that have been retracted, please include the rationale for doing so in the manuscript text, or remove these references and replace them with relevant current references. Any changes to the reference list should be mentioned in the rebuttal letter that accompanies your revised manuscript. If you need to cite a retracted article, indicate the article’s retracted status in the References list and also include a citation and full reference for the retraction notice.

Additional Editor Comments :

PLOS ONE Editorial Decision

Manuscript Number: PONE-D-25-52492R2

Title: Muslim religious leaders' views of climate change: An international survey

Authors: Bowen et al.

Editor: Dr. Morufu Olalekan Raimi

DECISION: Minor Revision Required

SUMMARY ASSESSMENT

I have now completed the evaluation of your second revised manuscript (R2) and the accompanying point-by-point response to my previous decision letter. You have made commendable progress in addressing several of the core concerns, particularly regarding:

• Explicit framing of the sample as exploratory and non-representative

• Clarification that analyses focus on pooled estimates only

• Addition of confidence intervals for Cronbach’s alpha

• Provision of complete PCA details in supplementary materials

• Revision of claims about Islamic teachings with appropriate citations

• Addition of a synthesized Conclusion section

However, three specific issues remain incompletely resolved from my prior decision letter. These are not fatal, but they require attention before final acceptance.

PERSISTING CONCERNS REQUIRING MINOR REVISION

1. Translation validation – limitations section remains insufficiently expanded

You correctly clarify that the survey did not include items referencing Quranic verses or principles such as Khalifah/Mizan (those emerged from open-ended responses). You also note that single-item measures preclude latent variable modeling. These clarifications are helpful.

Nevertheless, your expanded limitations text (page 27, lines 388-395) still states: “although the items were relatively straightforward and seem unlikely to vary substantially across cultural contexts.” This assumption remains methodologically unsupported. Concepts such as “climate change causes,” “willingness to promote,” and “government spending on technologies” are not self-evidently invariant across Arabic, French, Russian, Turkish, and English.

Required revision: Remove the phrase “and seem unlikely to vary substantially across cultural contexts” or replace it with a more neutral acknowledgment that cross-linguistic equivalence was not tested. The current wording downplays a genuine limitation.

2. Mode-region confounding – sensitivity analyses still not conducted, and justification remains problematic

You decline to conduct stratified or sensitivity analyses, citing insufficient power and small subgroup cell sizes. I accept that formal subgroup comparisons are not feasible. However, you also state that “mode-related differences may have introduced additional measurement error into the pooled estimates” (page 28, lines 403-405). This is an important acknowledgment, but it is presented as a speculative limitation rather than examined in any way.

Required revision: Please add a single paragraph in the Results or Limitations section that presents the overall mean scores for key dependent variables (e.g., willingness to promote mitigation actions, perceived importance of environment) separately for paper and online respondents without claiming statistical significance or regional inference. Present these as raw descriptive values only, accompanied by a clear caveat that mode is confounded with region. This simple transparency step would allow readers to assess the potential magnitude of mode-related differences.

If you remain unwilling to provide even descriptive unadjusted comparisons, you must state explicitly that the confounding of mode with region prevents any descriptive or inferential comparison, and remove the statement that mode-related differences “may have introduced additional measurement error” (since that cannot be evaluated).

3. Theoretical framing – minor clarification still needed

You clarify that the manuscript does not name elite cue theory, moral foundations theory, or value-belief-norm theory explicitly. I have verified this: the manuscript cites empirical and theoretical work related to these frameworks without labeling them as such. That is acceptable.

However, on page 3, lines 46–55, you write: “Theoretical and empirical work suggests that individuals' climate beliefs and behavior are influenced by their morals… values… and the norms and shared beliefs of the groups with which they identify.” You then state (page 4, lines 78-82) that the study is descriptive and exploratory, not designed to test theoretical predictions.

Required revision: Add a single bridging sentence after line 82, such as: “We therefore do not test specific predictions from these theoretical frameworks but instead provide an initial empirical assessment of Muslim religious leaders’ climate-related views.” This eliminates any ambiguity about the study’s theoretical ambitions.

ADDITIONAL MINOR ITEMS

• Abstract: The phrasing “Nearly all respondents agreed… and about half agreed…” remains slightly redundant but is acceptable. No change required.

• Conclusion: The new Conclusion section is appropriate. Well done.

FINAL DECISION AND NEXT STEPS

The manuscript addresses an important, under-researched topic. The R2 revisions have substantially improved transparency and methodological humility. With the three minor revisions specified above, the manuscript will meet PLOS ONE’s publication standards.

I invite you to submit a revised manuscript (R3) addressing the three points above.

Please provide a clean version and a tracked-changes version, along with a brief point-by-point response.

I expect to render a final decision without additional external review, provided the revisions are satisfactory.

Sincerely,

Dr. Morufu Olalekan Raimi

Academic Editor, PLOS ONE

[Note: HTML markup is below. Please do not edit.]

[NOTE: If reviewer comments were submitted as an attachment file, they will be attached to this email and accessible via the submission site. Please log into your account, locate the manuscript record, and check for the action link "View Attachments". If this link does not appear, there are no attachment files.]

To ensure your figures meet our technical requirements, please review our figure guidelines: https://journals.plos.org/plosone/s/figures

You may also use PLOS’s free figure tool, NAAS, to help you prepare publication quality figures: https://journals.plos.org/plosone/s/figures#loc-tools-for-figure-preparation.

NAAS will assess whether your figures meet our technical requirements by comparing each figure against our figure specifications.

# Author comment: Author response to Decision Letter 3

**DOI:** 10.1371/journal.pone.0353201.r006

11 Jun 2026

Please see attached file.

Attachment Submitted filename: response to editor.docx

# Editor report: Decision Letter 3

**DOI:** 10.1371/journal.pone.0353201.r007

**Authors:** Morufu Raimi

21 Jun 2026

Muslim religious leaders’ views of climate change: An international survey

PONE-D-25-52492R3

Dear Authors,

We’re pleased to inform you that your manuscript has been judged scientifically suitable for publication and will be formally accepted for publication once it meets all outstanding technical requirements.

Within one week, you’ll receive an e-mail detailing the required amendments. When these have been addressed, you’ll receive a formal acceptance letter and your manuscript will be scheduled for publication.

An invoice will be generated when your article is formally accepted. Please note, if your institution has a publishing partnership with PLOS and your article meets the relevant criteria, all or part of your publication costs will be covered. Please make sure your user information is up-to-date by logging into Editorial Manager at Editorial Manager® and clicking the ‘Update My Information' link at the top of the page. For questions related to billing, please contact billing support.

If your institution or institutions have a press office, please notify them about your upcoming paper to help maximize its impact. If they’ll be preparing press materials, please inform our press team as soon as possible -- no later than 48 hours after receiving the formal acceptance. Your manuscript will remain under strict press embargo until 2 pm Eastern Time on the date of publication. For more information, please contact onepress@plos.org.

Kind regards,

Morufu Olalekan Raimi, Ph.D

Academic Editor

PLOS One

Additional Editor Comments (optional):

PLOS ONE Editorial Decision

Manuscript Number: PONE-D-25-52492R3

Title: Muslim religious leaders' views of climate change: An international survey

Authors: Bowen et al.

Editor: Dr. Morufu Olalekan Raimi

DECISION: Accept

DETAILED ASSESSMENT

I have completed the evaluation of the revised manuscript (R3) and the accompanying point-by-point response to my previous decision letter. The authors have addressed all concerns raised in the R2 review thoroughly and appropriately.

RESOLUTION OF PREVIOUS CONCERNS

1. Translation Validation: The authors have correctly removed the problematic phrase "and seem unlikely to vary substantially across cultural contexts." The limitations section now appropriately acknowledges that cross-linguistic equivalence was not tested, removing the unsupported assumption that previously downplayed this genuine limitation.

2. Mode-Region Confounding: The authors have taken a methodologically rigorous approach. They explicitly state that the confounding of survey mode with geographic region prevents meaningful descriptive or inferential comparisons. They have removed the speculative statement about mode-related measurement error and provided descriptive statistics by survey mode in the Supporting Information with a clear caveat against interpretation as mode effects. This is transparent and appropriate given the study's design constraints.

3. Theoretical Framing: The bridging sentence added to the Introduction appropriately clarifies the descriptive, exploratory nature of the study. The authors now clearly state that they contextualize the study using existing theoretical frameworks without testing specific predictions from them.

MANUSCRIPT STRENGTHS

The manuscript addresses an important gap in the literature on religious leaders' climate views, providing rare empirical data from a geographically diverse sample of Muslim religious leaders. The authors demonstrate appropriate methodological humility throughout, consistently framing findings as exploratory and acknowledging limitations regarding generalizability. The study makes a valuable contribution to understanding the potential role of religious leaders in climate action.

MANUSCRIPT WEAKNESSES (already appropriately acknowledged)

The convenience sampling strategy, lack of back-translation, and confounding of survey mode with region are genuine limitations that the authors now address transparently. The small sample size precludes subgroup analyses and limits generalizability.

FINAL RECOMMENDATION

The authors have satisfactorily addressed all concerns from the previous review round. The manuscript is methodologically transparent, appropriately cautious in its claims, and makes a meaningful contribution to an under-researched topic. It meets PLOS ONE's publication standards.

I recommend acceptance of this manuscript in its current form.

Sincerely,

Dr. Morufu Olalekan Raimi

Academic Editor, PLOS ONE

# Editor report: Acceptance letter

**DOI:** 10.1371/journal.pone.0353201.r008

**Authors:** Morufu Raimi

PONE-D-25-52492R3

PLOS One

Dear Dr. Bowen,

I'm pleased to inform you that your manuscript has been deemed suitable for publication in PLOS One. Congratulations! Your manuscript is now being handed over to our production team.

At this stage, our production department will prepare your paper for publication. This includes ensuring the following:

* All references, tables, and figures are properly cited

* All relevant supporting information is included in the manuscript submission,

* There are no issues that prevent the paper from being properly typeset

You will receive further instructions from the production team, including instructions on how to review your proof when it is ready. Please keep in mind that we are working through a large volume of accepted articles, so please give us a few days to review your paper and let you know the next and final steps.

Lastly, if your institution or institutions have a press office, please let them know about your upcoming paper now to help maximize its impact. If they'll be preparing press materials, please inform our press team within the next 48 hours. Your manuscript will remain under strict press embargo until 2 pm Eastern Time on the date of publication. For more information, please contact onepress@plos.org.

You will receive an invoice from PLOS for your publication fee after your manuscript has reached the completed accept phase. If you receive an email requesting payment before acceptance or for any other service, this may be a phishing scheme. Learn how to identify phishing emails and protect your accounts at https://explore.plos.org/phishing.

If we can help with anything else, please email us at customercare@plos.org.

Thank you for submitting your work to PLOS One and supporting open access.

Kind regards,

PLOS ONE Editorial Office Staff

on behalf of

Prof Morufu Olalekan Raimi

Academic Editor

PLOS One
